# Sequencing a *Juglans regia* × *J. microcarpa* hybrid yields high-quality genome assemblies of parental species

**DOI:** 10.1038/s41438-019-0139-1

**Published:** 2019-03-25

**Authors:** Tingting Zhu, Le Wang, Frank M. You, Juan C. Rodriguez, Karin R. Deal, Limin Chen, Jie Li, Sandeep Chakraborty, Bipin Balan, Cai-Zhong Jiang, Patrick J. Brown, Charles A. Leslie, Mallikarjuna K. Aradhya, Abhaya M. Dandekar, Patrick E. McGuire, Daniel Kluepfel, Jan Dvorak, Ming-Cheng Luo

**Affiliations:** 1 Department of Plant Sciences, University of California, Davis, CA 95616 USA; 2 Ottawa Research and Development Centre, Agriculture and Agri-Food Canada, Ottawa, ON K1A 0C6 Canada; 3 Genome Center, University of California, Davis, CA 95616 USA; 4 Crops Pathology and Genetics Research Unit, USDA-ARS, Davis, CA 95616 USA; 5 National Clonal Germplasm Repository, USDA-ARS, Davis, CA 95616 USA

**Keywords:** Plant genetics, Plant evolution

## Abstract

Members of the genus *Juglans* are monecious wind-pollinated trees in the family Juglandaceae with highly heterozygous genomes, which greatly complicates genome sequence assembly. The genomes of interspecific hybrids are usually comprised of haploid genomes of parental species. We exploited this attribute of interspecific hybrids to avoid heterozygosity and sequenced an interspecific hybrid *Juglans microcarpa* × *J. regia* using a novel combination of single-molecule sequencing and optical genome mapping technologies. The resulting assemblies of both genomes were remarkably complete including chromosome termini and centromere regions. Chromosome termini consisted of arrays of telomeric repeats about 8 kb long and heterochromatic subtelomeric regions about 10 kb long. The centromeres consisted of arrays of a centromere-specific *Gypsy* retrotransposon and most contained genes, many of them transcribed. *Juglans* genomes evolved by a whole-genome-duplication dating back to the Cretaceous-Paleogene boundary and consist of two subgenomes, which were fractionated by numerous short gene deletions evenly distributed along the length of the chromosomes. Fractionation was shown to be asymmetric with one subgenome exhibiting greater gene loss than the other. The asymmetry of the process is ongoing and mirrors an asymmetry in gene expression between the subgenomes. Given the importance of *J. microcarpa* × *J. regia* hybrids as potential walnut rootstocks, we catalogued disease resistance genes in the parental genomes and studied their chromosomal distribution. We also estimated the molecular clock rates for woody perennials and deployed them in estimating divergence times of *Juglans* genomes and those of other woody perennials.

## Crop genetics: lessons from hybrids

The genetic sequence of a hybrid walnut tree sheds light on the evolution of the parental species and on some important traits. A study led by Ming-Cheng Luo and Jan Dvorak at the University of California Davis, USA, describes a new approach for producing high-quality genome assemblies of the parental species from interspecific hybrids. By applying long-read sequencing technology and optical mapping to a popular hybrid between cultivated Persian/English walnut and a wild relative that is native to North America they were able to completely assemble the genomes of these two species, gain insights into the evolution of their chromosomes and the distribution of disease-resistant genes. This approach could be used on other outcrossing  crops to generate reference-quality genome assemblies and accelerate genetic improvements in commercially important crops.

## Introduction

Haplotype phasing^1^ and navigating between allelic and nonallelic variation are the major challenges in assembling genomes of outcrossing species with high levels of heterozygosity such as found in members of the genus *Juglans*. Genome sequencing targeting outcrossing plants employed inbred lines^2^, haploids^3^, and megagametophytes^4^ to avoid heterozygosity.

Interspecific hybrids offer another strategy to avoid heterozygosity. Since the genome of an interspecific hybrid is usually comprised of haploid genomes of the parental species, interspecific hybrids have the same advantages for genome sequencing as haploids, but are usually easy to produce. Technical difficulties with allocating scaffolds to parental genomes have precluded the deployment of hybrids in genome sequencing. Using an interspecific hybrid between cultivated walnut and its wild relative *J. microcarpa*, we describe here a novel approach to sequencing hybrid genomes which results in a cost-effective high-quality genome assembly for both parents.

The cultivated Persian/English walnut, *Juglans regia*, is native to Asia whereas *J. microcarpa* is native to North America, where it occurs in riparian areas in the southwestern USA. Both species are wind-pollinated, highly heterozygous, and intolerant of inbreeding. Their hybrids are infertile. Both have a genome size of about 600 Mb^5^ with *n* = 16.

English walnut is an important nut crop with 3.8 million tons harvested worldwide in 2017 (http://www.fao.org/faostat/en/#data/QC). Walnut production has been steadily increasing in part due to health benefits derived from including walnuts in the human diet^6–8^. In the USA, English walnut trees are grown commercially using rootstocks chosen for their ability to tolerate such soil-borne pathogens as *Phytophthora* spp.^9^, lesion nematodes, and *Agrobacterium tumefaciens*^10^, which are all serious pathogens of walnut. The commercial hybrid rootstock *J. microcarpa* × *J. regia* possessing tolerance to soil borne diseases is extensively used in walnut production in California. The development of genomic resources for walnut and its wild relatives, including reference-quality genome sequences, will accelerate genetic improvement of walnut scions and rootstocks.

*Juglans* and its relatives in the family Juglandaceae are members of order Fagales, which includes many important forest trees. Reference-quality genome sequences will facilitate comparative genomics and will advance biology of this important group of woody perennials. Recent attempts to sequence the heterozygous genome of English walnut using traditional approaches resulted in assemblies with 4402 scaffolds with N50 = 640 kb^5,11^ and 25,670 scaffolds with N50 = 310 kb^12^. An attempt to sequence the heterozygous genome of *J. microcarpa* resulted in an even more fragmented assembly with 329,873 scaffolds with N50 = 136 kb^5^. The novel sequencing approach described here exploits the synergy between long-read sequencing and optical genome mapping. The average length of reads produced with long-read platforms exceeds the lengths of a vast majority of plant long-terminal-repeat retrotransposons (LTR-RTs), which results in a dramatically improved sequence assembly^13^. Contigs or scaffolds assembled from long-reads are sufficiently long to be aligned on genome-wide optical maps, which can be assembled with very high accuracy, even for large or polyploid plant genomes^14,15^. Alignments of the optical maps of a hybrid onto the optical maps of its parents will assign contigs to parental genomes^16^, while also serving as an assembly quality control^14^.

We used the assembled *J. regia* and *J. microcarpa* genome sequences in conjunction with the Juglandoid whole-genome-duplication (WGD)^17^, and the Juglandaceae fossil record to calibrate the molecular clock rate for woody perennials. We used the calibrated molecular clock to estimate the time of divergence of *Juglans* species and other woody perennials. Based on synteny within *Juglans* genomes, we allocated the 16 *Juglans* chromosomes produced by the Juglandoid WGD into eight homoeologous chromosome pairs and analyzed their evolution. Finally, we exploited the contiguity of the assemblies in the analyses of the structure and evolution of *Juglans* telomeres and centromeres and the distribution of disease resistance genes in the *J. regia* and *J. microcarpa* genomes.

## Results

### Genome assembly

We sequenced 58 Pacific Biosciences SMRT cells of the hybrid (MS1-56) *J. microcarpa* 31.01 (henceforth 31.01) × *J. regia* cv Serr (henceforth Serr) and assembled the reads (Table S1 and Fig. S1) into 460 polished contigs (for a flowchart of the entire assembly see Fig. S2). The total length of this assembly (MS1-56_v0) was 1,056,053,408 bp (N50 = 7,963,037 bp) (Table S2).

Next, we constructed two optical maps for the hybrid and one for each of its parents (Table S3). The N50 of the optical contigs ranged from 1.31 to 2.90 Mb. The parental maps consisted of ‘haploid’ regions, in which the haplotypes were similar enough to collapse into a single contig, and ‘diploid’ regions, in which the haplotypes were dissimilar enough to be assembled into separate contigs (phased). The haploid and diploid regions were identified by a map self-alignment (Fig. S3). The mean length across the phased regions was 108 Mb (16.8%) in Serr and 33 Mb (5.9%) in 31.01 (Table S4). Due to the inclusion of phased haplotypes in a map, the sum of the total lengths of the Serr and 31.01 optical maps was 13.4% longer than the length of the MS1-56 optical map (Fig. S3).

To ascertain whether the genome of our hybrid was complete, we compared the total length of its optical map to the sum of the lengths of the optical maps of the parents, which we edited to disregard the redundant haplotypes from the phased regions (Fig. S3). The total length of the edited maps of Serr and 31.01 differed from the length of the map of MS1-56 by only 1 Mb (0.09%) (Table S4). We therefore concluded that the hybrid genome was a complete representation of the parental genomes.

We aligned the sequence contigs on the optical map of the hybrid, stitched them into scaffolds (assembly MS1-56_scf, total length = 1,066,408,726 bp, N50 = 34,776,948 bp), and allocated 40 of them comprising 99.85% of the hybrid genome assembly into the parental genomes with the aid of the optical maps of the parents (Fig. S3). The remaining 224 scaffolds representing 0.15% of the hybrid genome sequence were too short (mean = 7.2 kb) to be aligned on an optical map and were aligned on Illumina sequence of the *J. regia* cv Chandler^11^ for assigning to parental genomes.

Finally, we ordered and oriented scaffolds on high-density genetic maps producing 16 pseudomolecules for each of the two genomes. They had only five gaps of unknown lengths (Fig. S4); the remaining gaps were estimated based on the optical map alignments. We then reduced gap lengths or closed them entirely with unassigned contigs or contigs produced with the 10X Genomics technology (Table S1).

We mapped 89X Illumina reads of the hybrid to the genome assemblies and detected 48,717 and 51,789 indels and 748 and 1,205 base substitutions in the Serr and 31.01 assemblies, respectively (about one error per 10,000 bp). We subsequently corrected these errors and produced the final assemblies JrSerr_v1.0 and Jm31.01_v1.0 (Table S5).

### Transposable element and gene annotation

**Fig. 1:**
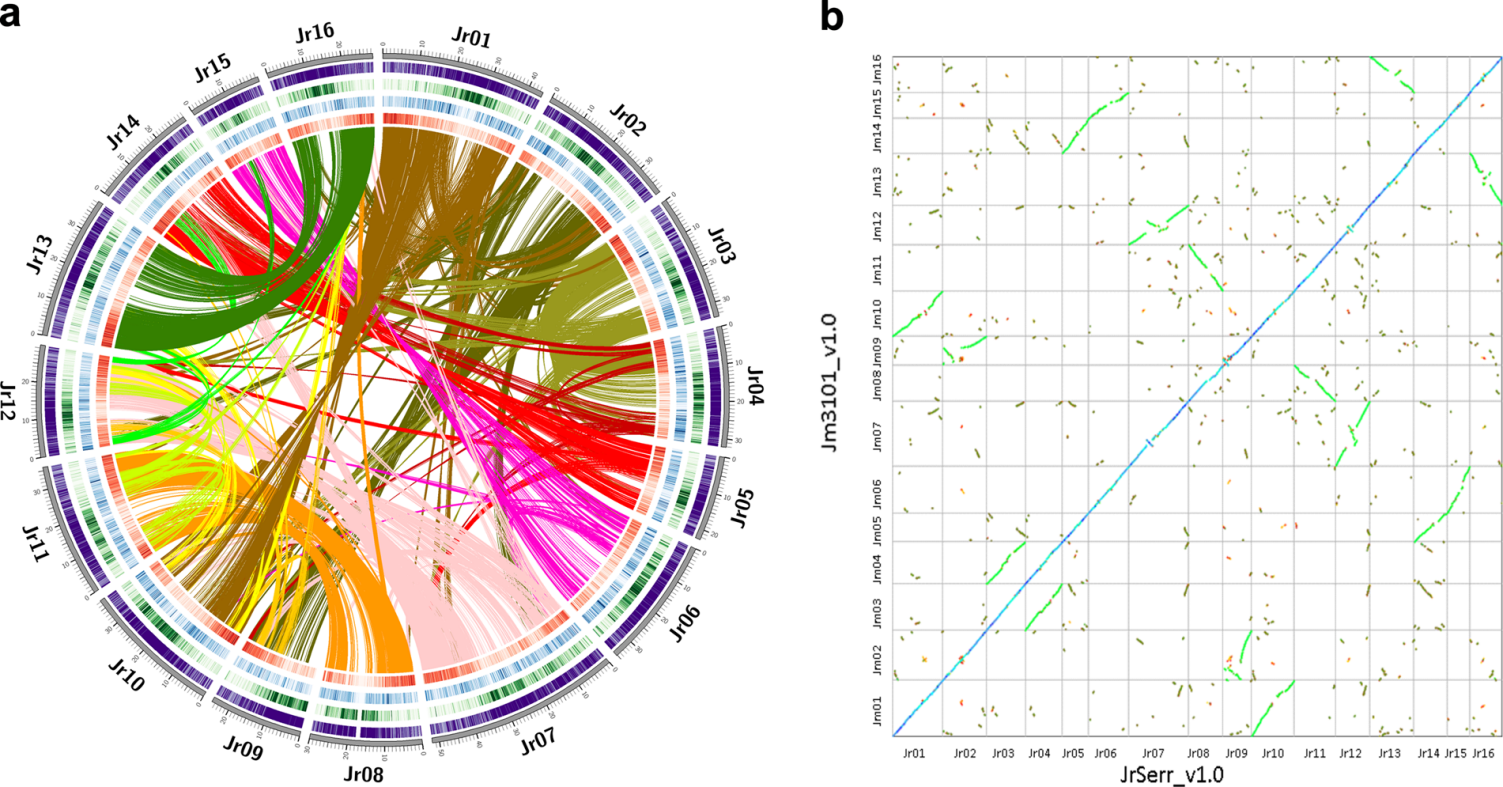
Structure and phylogeny of *Juglans* genomes. **a** Circular plot of the *Juglans regia* pseudomolecules. Circles from outside to inside are: pseudomolecule coordinates in Mb, density of all types of LTR-RTs, density of *Gypsy* LTR-RTs, density of *Copia* LTR-RTs, and gene density. Central lines connect syntenic blocks across chromosomes. Different colors represent different homoeologous chromosome pairs derived from the Juglandoid WGD. **b** Dot-plot alignments of the JrSerr_v1.0 pseudomolecules against Jm31.01_v1.0 pseudomolecules. The starts of the pseudomolecules are to the left or bottom. The blue diagonal across the plots of the 16 chromosomes indicate collinearity of homoeologous chromosomes between the two genomes. The green dots indicate homoeologous chromosomes within a genome, which originated by the Juglandoid WGD. The short black and red dot traces indicate paralogues produced the γ whole genome triplication

Transposable elements (TEs) represented 41.02 and 40.86% of the JrSerr_v1.0 and Jm31.01_v1.0 assemblies, respectively (Table S6). The density of the *Copia* LTR-RTs decreased and that of the *Gypsy* LTR-RTs increased from the chromosome ends towards the centromeres (Fig. 1a and S5). The JrSerr_v1.0 and Jm31.01_v1.0 assemblies each contained 0.03% satellite DNA and 351,359 and 349,951 simple sequence repeats (SSRs), respectively (Table S7). SSR density was highest in the distal regions of the pseudomolecules (Fig. S6).

We annotated 31,425 and 29,496 high-confidence (HC) protein-coding genes in the JrSerr_v1.0 and Jm31.01_v1.0 assemblies**;** 31,286 and 29,397 were on the pseudomolecules and 139 and 99 were on unassigned scaffolds (Table S8, S9). Of 1,440 BUSCO v3 genes, 96.0 and 95.2% were among the HC genes annotated in the JrSerr_v1.0 and Jm31.01_v1.0 assemblies, which is comparable to coverage reported in other genome annotations in plants (Table S10). Serr and 31.01 genes were on average 4,226 and 4,391 bp long and had 5.44 and 5.60 exons averaging 258.64 and 249.38 bp (Table S8). Transcription factors were encoded by 2,046 (6.5%) and 2,032 (6.9%) genes in the JrSerr_v1.0 and Jm31.01_v1.0 assemblies (Table S11). There were 18,772 (59.7%) and 18,891 (64.0%) single-copy genes and 744 and 625 multigene families in the JrSerr_v1.0 and Jm31.01_v1.0 assemblies. The larger number of genes annotated in the *J. regia* genome compared to the *J. microcarpa* genome was due to the larger number of duplicated genes in the former.

Average gene density was one gene per 16.9 and 17.7 kb in the JrSerr_v1.0 and Jm31.01_v1.0 pseudomolecules. Gene density decreased and TE density increased from pseudomolecule ends to centromeric regions (Fig. 1a and S5), and the two variables were negatively correlated (*r* = −0.82, *p* < 0.01). Gene density was positively correlated with meiotic recombination rates^17^ along the *J. regia* pseudomolecules (*r* = 0.78, *p* < 0.01).

### *Juglans* genome structure and evolution

Of the genes annotated on the Serr pseudomolecules, 26,403 (84.4%) were collinear with genes on the *J. microcarpa* pseudomolecules (File S1). The two genomes differed by 28 inversions involving > 3 collinear genes, 21 segmental duplications, 3 intra- and 14 inter-chromosomal interstitial translocations, but no terminal translocation (File S1, Fig. 1b). Only 90 (0.28%) of the JrSerr_v1.0 genes were not detected in the Jm31.01_v1.0 assembly.

**Fig. 2:**
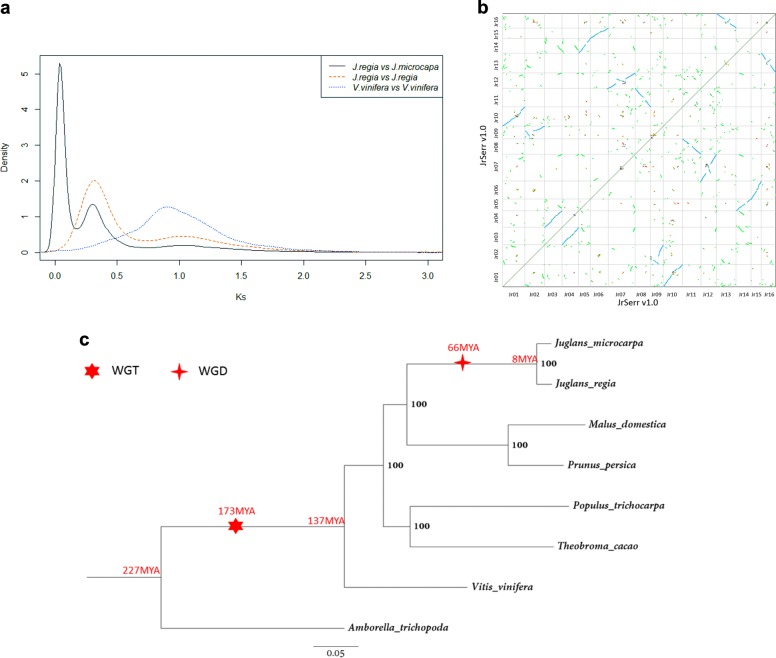
Whole-genome duplication and triplication in the *Juglans* lineage. **a** Densities of *K*s divergence in the BLASTP homology searches of JrSerr_v1.0 against Jm31.01_v1.0, within JrSerr_v1.0, and within the grape genome^18^. **b** Self-alignments of the *J. regia* Serr pseudomolecules. Pseudomolecules are oriented as in 1b. **c** Divergence of the genomes of *Vitis vinifera*, *Prunus persica*, *Amborella trichopoda*, *Populus trichocarpa, Theobroma cacao*, *Malus domestica*, *J. regia* cv Serr, and *J. microcarpa* acc. 31.01. The dendogram was constructed from *K*a divergence of 1155 single-copy genes. Bootstrap confidence on each node is shown. Also shown are estimated times of critical events in *Juglans* genome evolution, including the γWGT and the Juglandoid WGD (Table S13)

We computed *Ks* divergence among the *J. regia* and *J. microcarpa* genes to analyze gene duplication and divergence. The *Ks* plot showed three peaks (Fig. 2a). The first peak mostly consisted of *Ks* values between orthologous genes in the two genomes and reflected their divergence. The second peak coincided with the major *Ks* peak in self-searches within the Serr and 31.01 genomes and reflected the divergence of paralogous genes, which originated by the Juglandoid WGD. The third peak coincided with the major peak in self-searches within the grape (*Vitis vinifera*) genome and reflected divergence between genes duplicated by the whole genome triplication (γWGT) first described in the grape genome^18^. These inferences were confirmed by self-alignment plots (Figs. 1a, 2b, S5, File S1), a plot of the JrSerr_v1.0 pseudomolecules against the grape pseudomolecules (Fig. S7), and a plot against the *Amborella trichopoda* scaffolds^19^ (Fig. S8).

With the sole exception of *Rhoiptelea chiliantha*, all species of Juglandaceae share *n* = 16 or a chromosome number derived from it^20^. *Rhoiptelea chiliantha* and the neighboring family Myricaceae have *n* = 8; *n* = 16 was derived from *n* = 8 by the Juglandoid WGD^17^. The fossil record showed that Juglandaceae appeared in the upper Cretaceous and the family radiated in the Paleocene^21–23^. The Juglandoid WGD must have therefore originated prior to the radiation of Juglandaceae in the Paleocene but after the divergence of *R. chiliantha* in the Cretaceous. We therefore chose the Cretaceous-Paleogene (C-Pg) boundary (66 MYA) as the approximate time of the origin of the Juglandoid WGD. Of the 31,286 genes on the JrSerr_v1.0 pseudomolecules, 12,195 (39.0%) were genes duplicated by the Juglandoid WGD that were in collinear locations on the homoeologous chromosomes. Using the WGD time estimate and the average *Ka* and *Ks* between these paralogues in the Serr and 31.01 genomes, we estimated clock rates as 5.6 × 10^–10^ nucleotide substitutions year^−1^ using *Ka* and 2.5 × 10^–9^ nucleotide substitutions year^−1^ using *Ks* (Table S12).

To date the divergence of *J. regia* and *J. microcarpa*, we identified 1155 single-copy genes shared by *J. regia, J. microcarpa*, and six other sequenced genomes of woody perennials, including grape and *A. trichopoda*, and constructed a dendogram (Fig. 2c). Its topology was consistent with branching of the tree of life (http://tolweb.org/Core_Eudicots/20714). However, the dating of *J. regia* and *J. microcarpa* divergence, about 8 MYA, was one-half to one-quarter of previously reported estimates^5,24,25^. Our estimates of divergence time of the *Amborella* lineage, the grape lineage, and the origin of the γWGT (Table S13) would be more recent than the published data or fossil records^26–28^ if this discrepancy were caused by an incorrectly calibrated molecular clock or incorrectly computed divergence times. However, this was not the case.

If we failed to correctly phase the *J. regia* and *J. microcarpa* haplotypes during the assembly of the hybrid genome sequence, orthologous genes in our JrSerr_v1.0 and Jm31.01_v1.0 assemblies would appear more closely related to each other than they actually are and the age of divergence of the two genomes could be underestimated. To test this possibility, we computed the *Ka* and *Ks* values and time of divergence between *J. regia* and *J. microcarpa* using sequences of 1077 single-copy genes independently sequenced by Stevens et al.^5^. The *Ka* and *Ks* values we obtained were similar to those computed from our gene sequences (Table S13). We then constructed a chronogram including genomes of peach, apple, poplar, and cocoa and the six *Juglans* genomes reported by Stevens et al^5^. in addition to the genomes used in Fig. 2c (Fig. S9). The topology of the chronogram was similar to that in Fig. 2c and the divergence time estimates of the branches shared with Fig. 2c were within the 95% confidence intervals of estimates in Fig. 2c (Table S13). Based on these analyses, we suggest that the discrepancies in dating of the *J. regia* and *J. microcarpa* divergence is due to the use of an unrealistic rate of the molecular clock in the previous studies.

We analyzed gene collinearity along homoeologous chromosomes within the Serr genome and detected 38 paracentric inversions involving >3 collinear genes and 20 intra- and 16 inter-chromosomal interstitial translocations. The homoeologues did not differ by any terminal translocations and retained a 1:1 relationship. Using the grape genome as an outgroup, we assigned rearrangements to phylogenetic branches (File S1) and computed the rates of their accumulation. The rates ranged from 0.4 to 1.4 major rearrangements per MY (Table S14).

The collinearity analysis showed that the 16 *J. regia* chromosomes can be built from 142 major synteny blocks making up the 19 grape chromosomes. Of them, 122 (85.9%) were shared by the *J. regia* homoeologous chromosomes, and were in the same order along the them (File S1 and Table S15). These rearrangements must have taken place prior to the Juglandoid WGD. We found 43.7% of the *J. regia* genes collinear with genes on the grape pseudomolecules (Table S16).

### Asymmetric (biased) fractionation of the Juglandoid WGD

**Fig. 3:**
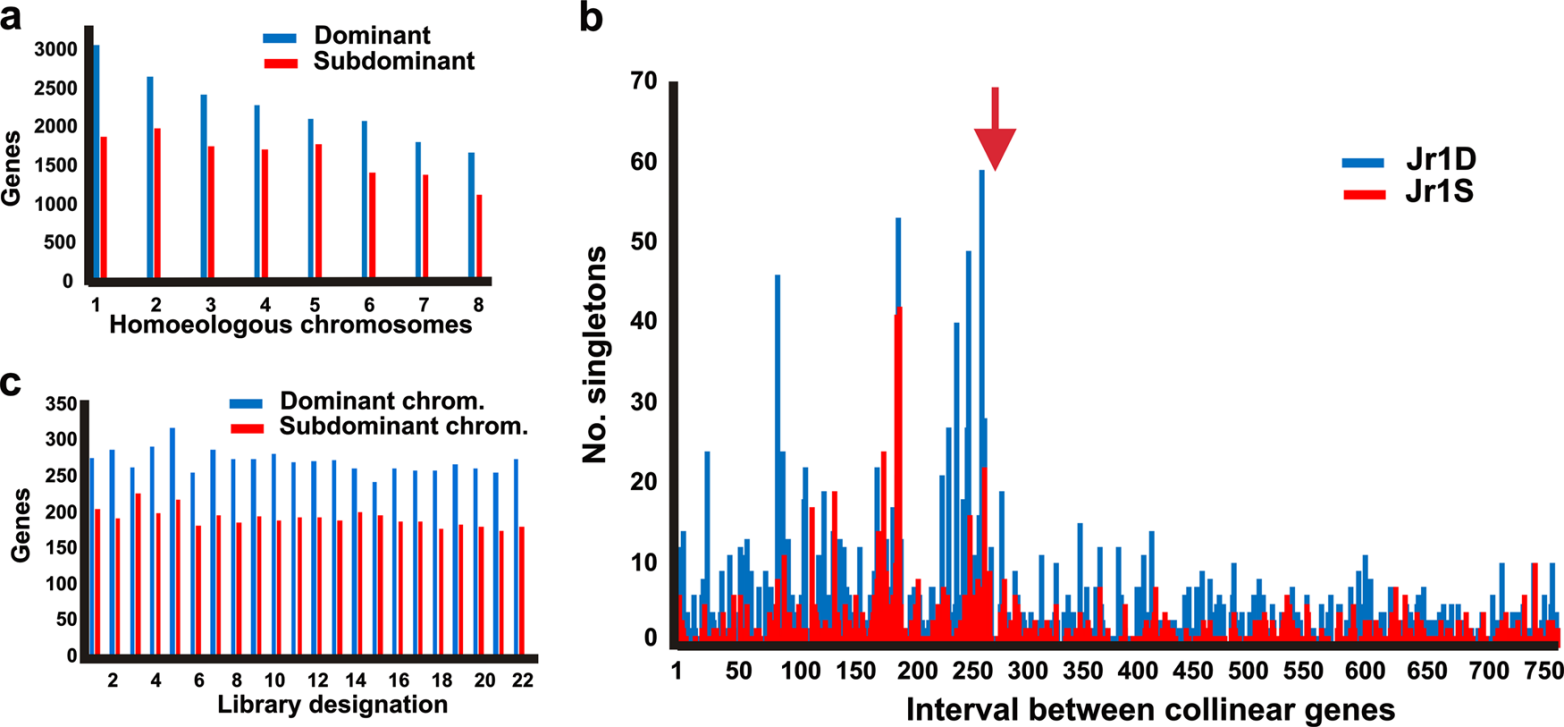
Asymmetric gene fractionation of the Juglandoid WGD. **a** Numbers of genes annotated on the JrSerr_v1.0 dominant and subdominant pseudomolecules. Numbers on the horizontal axis are designations of individual *Juglans* homoeologous groups, each consisting of one dominant (D) and one subdominant (S) chromosome. **b** Numbers of singleton genes in intervals delimited by conserved paralogues on the dominant chromosome Jr1D and subdominant homoeologues Jr1S. The homoeologous pseudomolecules Jr1D and Jr1S were subdivided into intervals delimited by successive pairs of paralogous genes in collinear locations on the homoeologues. The noncollinear genes (singletons) were counted in each interval (histogram bars) in the dominant pseudomolecule (blue) and subdominant pseudomolecule (red). The starting nucleotides of the pseudomolecules are to the left. The red arrow indicates the location of the centromere in the dominant pseudomolecules. **c** Numbers of genes on the pseudomolecule of the dominant chromosome Jr1D (blue) and subdominant chromosome Jr1S (red) showing dominant expression (two-fold higher expression than that of the orthologous gene) in 22 different *Juglans regia* RNAseq datasets. The description of the RNAseq datasets (coded at the horizontal axis of the graph) is in Fig. S12 and Table S20

In each pair of homoeologous chromosomes within a *Juglans* genome, one chromosome contained more genes than the other (Fig. 3a). We denoted the homoeologues with more genes as “dominant” and those with fewer genes as “subdominant,” allocated the 16 *Juglans* chromosomes into eight homoeologous chromosome pairs, which we arranged in descending order based on the number of genes in the dominant chromosome (Fig. 3a), and renamed the chromosomes (Table S17).

There were 18,179 and 17,093 genes in the dominant subgenome and 13,107 and 12,304 genes in the subdominant subgenome in JrSerr_v1.0 and Jm31.01_v1.0, respectively (Table S18). Both dominant and subdominant subgenomes had fewer genes than are in the *A. trichopoda* genome (26,846 genes)^19^, which was not subject to a recent WGD (Fig. 2c). This comparison indicates that both dominant and subdominant *Juglans* subgenomes have lost genes since the WGD.

The difference in gene loss between dominant and subdominant homoeologues was nearly constant among the homoeologous pairs (Fig. 3a), suggesting that the rate of gene loss was intrinsic to a subgenome. The rate of gene loss was also nearly constant along each chromosome. We subdivided homoeologous chromosomes into sections delineated by successive pairs of collinear genes and counted the numbers of singleton genes in the intervening intervals (Table S19). In the JrSerr_v1.0 pseudomolecules, 13,791 singletons were on the dominant chromosomes (mean of 3.2 per interval) but only 8,261 were on the subdominant chromosomes (mean of 1.9 per interval, *p* = 0.0001, paired *t*-test, *N* = 16). The numbers of singletons per interval varied little along each chromosome, except for the proximal regions, indicating that gene loss was uniform along the chromosomes and occurred by many deletions involving one or few genes along a chromosome (Fig. 3b and S10). In each chromosome, deletions were larger and more frequent in the proximal region than the rest of the chromosome, as the numbers of singletons per interval were greatly elevated in proximal regions. We also expressed the numbers of singletons per 2-Mb non-overlapping windows. Most of the windows in subdominant chromosomes contained fewer singletons than those in the dominant homoeologues (Fig. S11).

Since the divergence of the *J. regia* and *J. microcarpa* lineages 8 MYA, 28.7% of singletons were lost from the subdominant chromosomes in *J. microcarpa* but only 14.9% from the corresponding intervals in the dominant homoeologues (*p* = 0.002, two-tailed paired *t*-test). Thus, the factor that caused the asymmetry in fractionation has persisted in the subgenomes since their origin to the recent past. In 22 RNAseq datasets (Table S20), genes on the dominant chromosomes were on average transcribed significantly more than their paralogues on the subdominant chromosomes (paired *t*-test, *p* *<* 1.0 × 10^–8^) (Fig. 3c and S12, File S2).

### rRNA genes

We failed to assemble the sequences of the arrays of 18S-5.8S-26S rRNA genes, although we were able to assemble an optical map of the locus on Jr4S (Fig. S13). The locus was 1,310,076 bp long (16,646,858–17,956,934 bp) and contained 112 gene units repeated in tandem; only on unit was present in the pseudomolecule.

### Telomeres

**Fig. 4:**
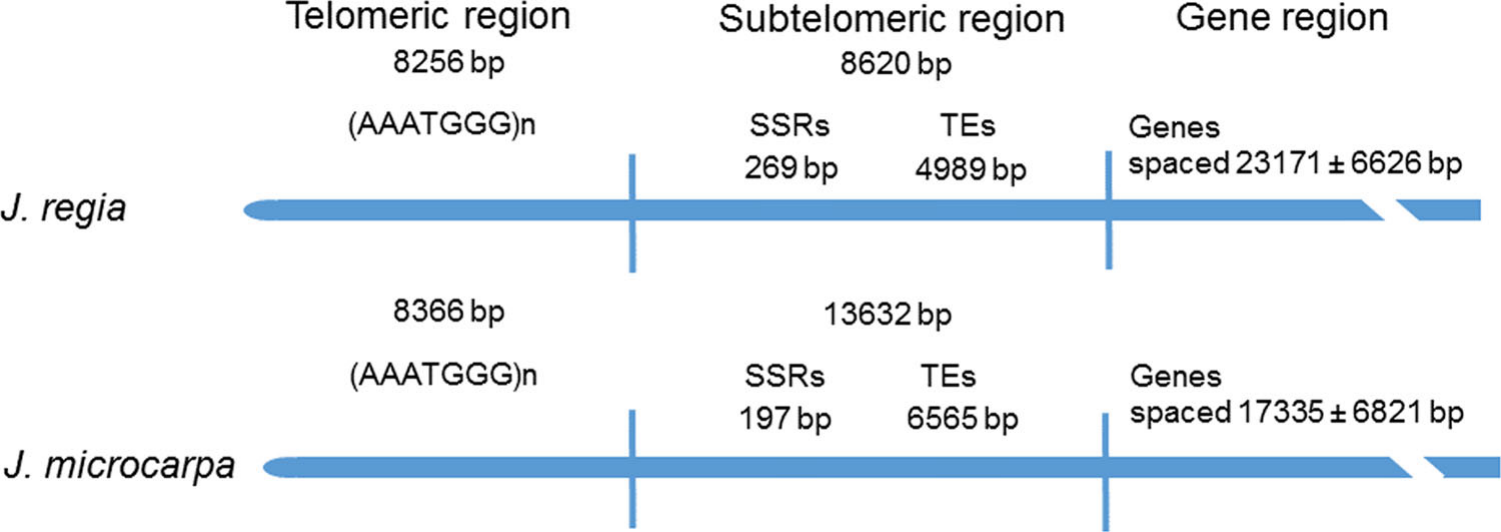
Structure of chromosome termini in the *Juglans regia* and *J. microcarpa* pseudomolecules. The telomeric region consists entirely of telomeric repeats. The subtelomeric region consists of satellite motifs and TEs. The gene region starts with the coding sequence of the first gene. Mean intergenic distances and 95% confidence intervals (*N* = 16 and 11 for *J. regia* and *J. microcarpa*, respectively) were computed for the first three protein-coding genes

Complete arrays of the telomeric repeat CCCTAAA were present at 16 *J. regia* and 11 *J. microcarpa* termini (Table S21). These termini had a tripartite structure (Fig. 4). The telomeric regions consisted of monotonous arrays of telomeric repeats, with a sole exception of Jm6S which included a 47-bp (T)_n_ SSR (File S3). The subtelomeric region spanned the distance from the last telomeric repeat to the first gene and had the characteristics of heterochromatin by consisting of various microsatellites interspersed with TEs. Juxtaposed to the subtelomeric region was the euchromatic region containing genes. Spacing of genes was estimated for the three most distal genes and was similar to the genome average (Fig. 4).

We anticipated finding a transitional zone containing degraded telomeric repeats near the boundaries between the telomeric and subtelomeric regions. However, the boundaries were almost always sharp; the last perfect telomeric repeat was juxtaposed to unrelated sequences in the subtelomeric region (File S4). This pattern would emerge if the entire telomeric region was occasionally forged by the telomerase de novo in the germline.

### Centromeres

**Fig. 5:**
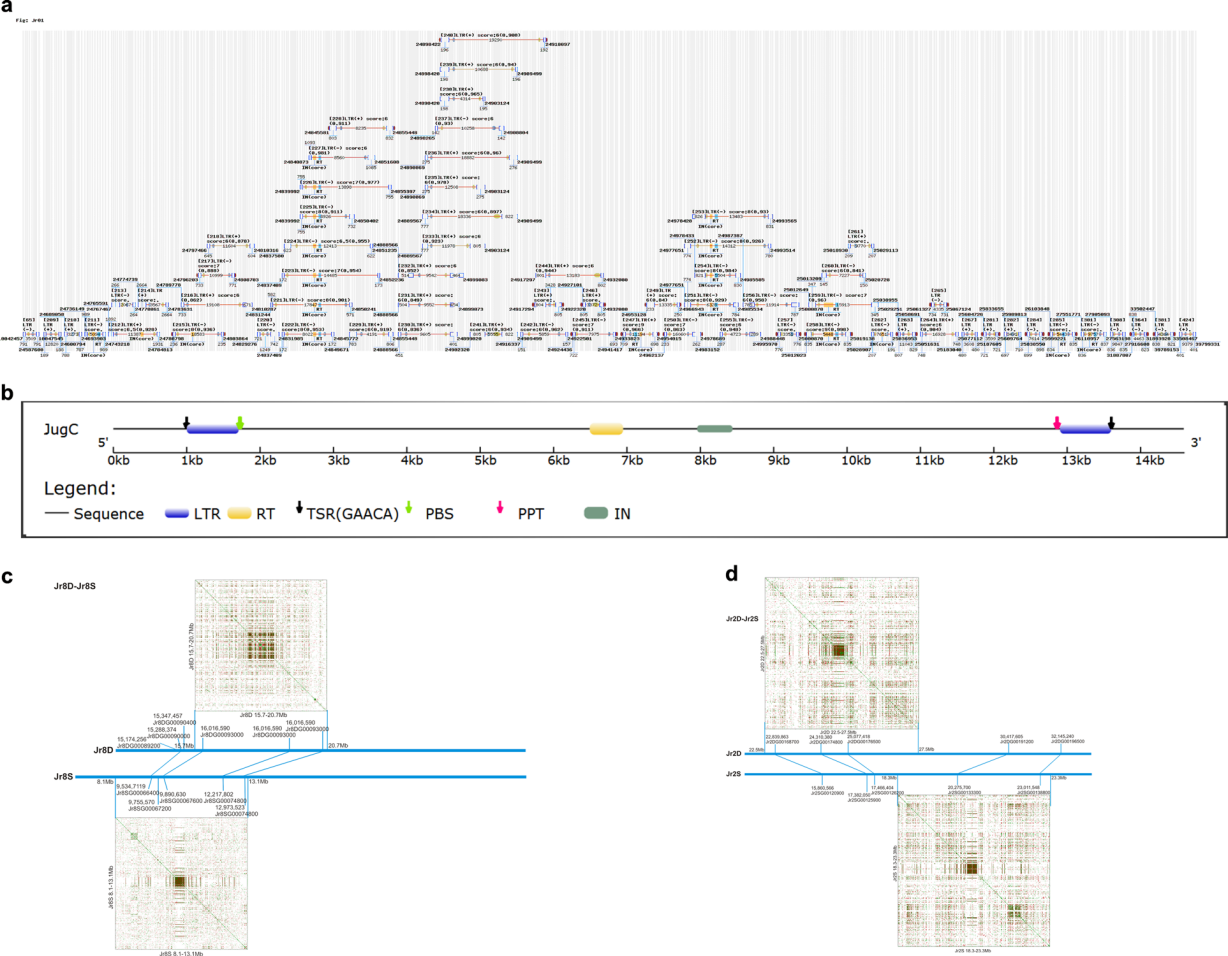
Centromere structure and evolution. **a** Distribution of the *JCR* repeats in the centromeric region of Jr2D. **b** Structure of the centromeric *JCR Gypsy* element. **c** and **d** Structural comparisons of 5-Mb intervals including the *JCR* arrays between *J. regia* homoeologous chromosome pairs Jr8D-Jr8S and Jr2D-Jr2S. Lines connect collinear loci in homoeologous chromosomes. The coordinates of the edges of the 5-Mb intervals in Mb are shown. **c** The Jr8D-Jr8S homoeologous chromosome pair illustrating the conservation of the centromere location on homoeologous chromosomes. A *JCR* array is located in the interval Jr8DG00090000-Jr8DG00105900 on Jr8D. This interval corresponds to interval Jr8SG00067200-Jr8SG00077500 on Jr8S, which also contains a *JCR* array. **d** The Jr2D-Jr2S homoeologous chromosome pair illustrating the repositioning of the *JCR* array into different interval without perturbation of the order of collinear genes. In Jr2D, the *JCR* array is within interval Jr2DG00174800-Jr2DG00176500. This interval corresponds to interval Jr2SG00125900-Jr2SG00126200 in Jr2S, which is devoid of a JCR array. Instead, a JCR array is in interval Jr2SG00133300-Jr2SG00138800 in pseudomolecule Jr2S

There was one specific region greatly enriched for repeats and impoverished for genes in each *J. regia* (Fig. S14) and *J. microcarpa* pseudomolecule. These regions were at similar locations on the corresponding *J. regia* and *J. microcarpa* pseudomolecules, contained the same sequences, and had similar structure (Fig. S15). They coincided with the locations of the *J. regia* centromeres^17^. To further characterize these regions, we generated self-synteny dot-plots for a 5-Mb region surrounding each repeat peak. These analyses revealed the existence of clusters of a 12.5-kb *Gypsy* LTR-RT (Fig. 5a), which we name *JCR* (Juglandoid Centromeric Repeat) (Fig. 5b). *JCR*s were nearly exclusively located in the centromeres. For instance, we found 62 complete *JCRs* within a 3-Mb centromeric region of Jr2D (Fig. 5a), but only four copies in the remaining 41 Mb of that pseudomolecule. Analogous CRs of the *Ty/Gypsy* superfamily have been located in the centromere cores in grasses^29,30^, cotton^31^, soybean^32^, beet^33^, and *Brassica*^34^.

We located 403 protein-coding genes, many conserved and showing collinearity with genes in the grape genome, within the *JCR* arrays (File S1). For 201 of them, we found transcripts in two or more of the 22 RNAseq datasets we analyzed, indicating that many of these genes are expressed (File S5).

We used genes located within and near the *JCR* arrays and collinear on Serr dominant and subdominant homoeologous pseudomolecules as landmarks for comparisons of the locations of homoeologous centromeres within the Serr pseudomolecules (Figs. 5c, d). In the homoeologous chromosome pairs Jr4D-Jr4S, Jr5D-Jr5S, and Jr8D-Jr8S (Fig. 5c), the *JCR* array was located in corresponding intervals (Fig. S16). In the remaining pairs, the *JCR* array was located in differing locations. In the Jr2D-Jr2S pair the array was repositioned to a new location without perturbing the order of the collinear genes (Fig. 5d). In that pair, centromere repositioning likely occurred by an intrachromosomal translocation of the *JCR* array into a new location. In homoeologous chromosome pairs Jr1D-Jr1S, Jr6D-Jr6S, and Jr7D-Jr7S, the *JCR* array was repositioned to a new location by an inversion with one breakpoint within the *JCR* array (Fig. S16). If a break were at the edge of the JCR array, such an inversion may reposition the entire *JCR* block and the entire centromere. If the break were in the middle of the array, it would create pair of *JCR* arrays, as seen in Jr1S (Fig. S16). In this specific case, it needs to be determined which of the *JCR* arrays is the active centromere and which is silent.

We frequently found inversions to terminate in the centromeric regions, suggesting that centromeric regions were prone to rearrangements (File S1). The centromeric regions were also prone to gene transposition as shown by the comparison of gene collinearity between the JrSerr_v1.0 and Jm31.01_v1.0 pseudomolecules (Fig. S17).

### Disease resistance genes

Since *J. microcarpa* is important for the development of disease-resistant rootstocks for walnut commercial production, we paid special attention to resistance gene analogues (RGA, henceforth R genes) in the two genomes (File S6). There was very little difference in the number of R genes between a cultivated and a wild species. There were 942 R genes in JrSerr_v1.0 and 903 in Jm31.01_v1.0. The R genes represented 3% of the total number of genes in both genomes.

We classified the R genes into 11 categories (Table S22). The most frequent category was the receptor-like kinase (RLK), which represented 62 and 58% of the R genes in JrSerr_v1.0 and Jm31.01_v1.0 assemblies.

**Fig. 6:**
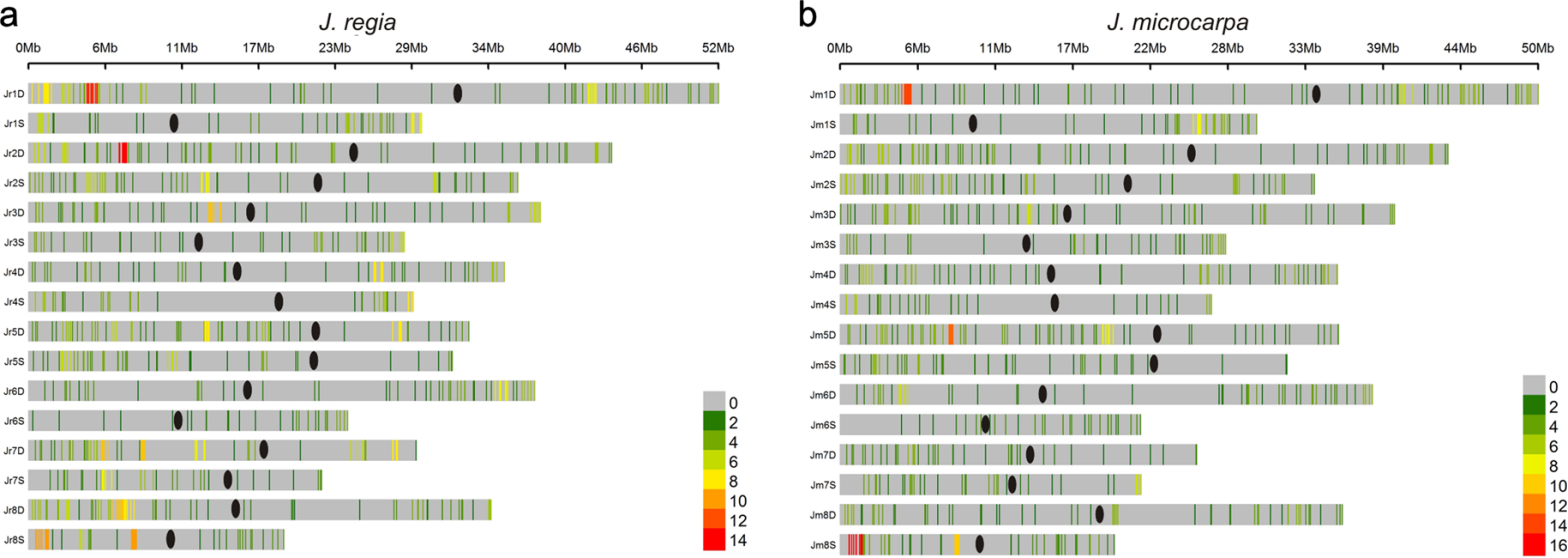
The distribution of the R genes along *J. regia* Serr (**a**) and *J. microcarpa* 31.01 (**b**) chromosomes. Heat maps show the numbers of R genes per 1 Mb non-overlapping windows along the pseudomolecules. The starting nucleotides of the pseudomolecules are to the left. Centromeres are indicated by black ovals

R genes were present on all chromosomes, but showed a tendency to be located in the distal regions (Fig. 6). We divided the total number of genes present in a chromosome arm into 20 windows containing equal numbers of genes and computed the proportion of R genes in each window. The proportions correlated with bin locations on the telomere-to-centromere axis in the JrSerr_v1.0 pseudomolecules (*r* = −0.48, *p* = 0.033) but in the Jm31.01_v1.0 pseudomolecules the correlation was not significant (*r* *=* −0.35, *p* = 0.095). The distribution of R gene was similar between homoeologous *J. regia* and *J. microcarpa* pseudomolecules, but, there was no similarity in their distribution between homoeologous chromosomes within a genome (Fig. 6).

Of the 942 R genes in the JrSerr_v1.0 assembly, 793 (84.2%) were in collinear location in the Jm31.01_v1.0 assembly. This was nearly identical to the 84.4% collinearity for the remaining 30,345 genes in the Serr genome. A total of 378 (40.1%) of the R genes were in collinear locations on the Serr homoeologous chromosomes. Of the remaining Serr genes, 39.0% were in collinear locations on the homoeologous chromosomes. Therefore, there appears to be no difference in dynamism of the R gene families and the rest of the *Juglans* genes.

## Discussion

### Sequencing approach

Sequencing of heterozygous *Juglans* genomes using the short-read technology resulted in assemblies with short contigs and scaffolds^5,11,12^. Similar results were previously obtained for other outcrossing woody perennials with the Sanger sequencing technology^18,35–37^, which is often considered as the gold standard of DNA sequencing. We attribute the great increase in the contiguity of our assemblies compared to the previous attempts to sequence *Juglans* genomes to the use of a long-read sequencing technology^38^ in addition to the haploid state of the *J. regia* and *J. microcarpa* genomes in the interspecific hybrid.

A well-known problem accompanying the long-read sequencing technologies is high error rates. These errors must be corrected during genome assembly. We were able to correct most of the errors during polishing of the assemblies and achieved a rate of one error in 10,000 nucleotides, which matched a standard recommended for the human genome for the Sanger sequencing technology^39^. In both genomes, 98% of errors that remained in the assembly after polishing were indels, indicating that the polishing algorithm was more efficient  in detecting base substitution errors than base indel errors. We corrected both types of errors by mapping Illumina reads into the polished assemblies.

Long contigs obtained with the PacBio long-read technology for the *J. microcarpa* × *J. regia* hybrid were critical for efficient aligning sequence contigs on the optical maps of the parental species and their allocation to parental genomes. We were able to allocate 99.85% of the hybrid genome sequence to the parental genomes. Contigs that are too short to be aligned on optical maps can be allocated to parental genomes by aligning them on Illumina reads of the parental genomes. In our case, we used the published Illumina sequence of *J. regia* cv Chandler^11^ for allocating short contigs to parental genomes.

The optical maps played additional roles in our genome sequence assembly. They facilitated construction of long scaffolds, which reached N50 = 34.8 Mb and approached or equaled the lengths of the chromosomes. They also facilitated detection of misassembled contigs and the estimation of the actual lengths of most of the gaps. By using two independently made optical maps for each genome (one for the hybrid and one for each parent) we were able to decide whether a discrepancy between an optical map and a sequence was due to an error in the sequence or in the map.

A synergy between long-read sequencing technology and optical mapping made it possible to leverage the haploid state of the genomes in the *J. microcarpa* × *J. regia* hybrid to produce remarkably complete genome assemblies. A concern about using a hybrid for the construction of a reference genome sequence is that in some instances one of the parental genomes is partially or completely eliminated from a hybrid. The *Hordeum vulgare* × *H. bulbosum* hybrids are the best known example^40^. Loss of 10% or more of DNA from a genome in artificial polyploids produced by the colchicine treatment of interspecific hybrids has been reported and may be another reason for concern^41–44^. To ascertain whether the genome of our hybrid was complete, we compared the total length of its optical map to the sum of the lengths of the optical maps of the parents. The maps differed by a mere 0.09%, which is below the error rate of these measurements. We therefore concluded that the hybrid genome was a complete representation of the parental genomes. The main source of genetic instability in hybrids and polyploids is meiosis^45^. Since DNA used for the construction of sequencing libraries and optical maps is isolated from young somatic tissues this cause of instability is irrelevant. Nevertheless, the possibility of somatic instability of an interspecific hybrid should be kept in mind and the additivity of the hybrid genomes should be checked. Making the use of the optical map trio, as we did, is the most practical approach, because it requires very little additional work.

### Juglans genome evolution

We showed that *Juglans* genomes consist of eight pairs of homoeologous chromosomes, which originated by the Juglandoid WGD. The chromosomes were subjected to a highly asymmetric fractionation. Similar asymmetric fractionation following WGD was described in other species^46–49^. In wheat, the larger B genome has lost twice as much DNA as the smaller A genome since the origin of tetraploid wheat about 0.3 MYA^47^. If a difference in subgenome sizes were the cause of asymmetric gene loss in walnut, the subgenomes should differ in gene density, since large genomes are less gene dense than small genomes. That was not the case. Gene density was similar in the dominant and subdominant homoeologues.

We showed that the asymmetric fractionation of *Juglans* subgenomes has not relented in the recent past. The factor that caused the asymmetry is therefore likely active in the current *Juglans* genomes. We suggest that the factor is asymmetric gene expression on dominant and subdominant chromosomes. An asymmetry in expression of genes on dominant and subdominant homoeologues has been reported in plant families as diverse as Poaceae^50,51^, Brassicaceae^48^, and Malvaceae^49^. A relevant question is what causes the asymmetry in gene expression. In *Arabidopsis* and *Brassica*, reduced gene expression was linked to the silencing effects of small, 24-nt RNAs amplified against specific LTR-RTs^52^. The methylation of LTR-RTs, and their contribution to the pool of siRNAs in walnut should be analyzed to determine if the same mechanism is the basis for the differences in gene expression in the *Juglans* subgenomes.

The rates of accumulation of structural changes in the *Juglans* genomes ranged from 0.4 to 1.4 major rearrangements per MY during the 66 MY of evolution. These rate estimates are among the lowest we have obtained to date^14,15^. Furthermore, 43.7% of the *J. regia* genes were collinear with genes on the grape pseudomolecules, which is remarkable considering that the two lineages diverged more than 100 MYA. This high gene collinearity among woody perennials contrasts with low gene collinearity in comparisons involving genomes of herbaceous plants, such as those of grasses (Table S16), and provide additional evidence for slow genome evolution in woody perennials^17^.

## Materials and methods

### Trees

DJUG 31.01 is a tree grown from *J. microcarpa* seeds collected near Sanderson TX USA by Loy Shreve as accession 83–131. Serr is the result of a cross between *J. regia* cv Payne of unknown seedling origin and PI 159568, a USDA plant introduction of *J. regia* from Paghman, Afghanistan. We generated clones of their hybrid MS1-56 by micropropagating the epicotyl excised from an immature hybrid embryo and transplanted rooted clones into pots in a greenhouse.

### Optical map construction

We collected leaves from tissue culture-grown clones for MS1-56 and orchard trees for Serr and 31.01 and provided them to Amplicon Express (Pullman WA USA) for high-molecular-weight (HMW) (>500 kb) DNA isolation. Based on the frequency of recognition sites in the genome sequence of *J. regia* cv Chandler^11^, we selected nicking endonucleases Nt.*Bsp*QI and Nb.*Bss*SI (New England BioLabs, Ipswich MA USA) for DNA nicking. We treated HMW DNA of MS1-56 separately with both enzymes and that of Serr and 31.01 only with Nt.*Bsp*QI. We stained nicked molecules according to the IrysPrep Reagent Kit instructions [Bionano Genomics, San Diego CA USA, (henceforth BNG)] and imaged the stained DNA molecules with the Irys system (BNG). To obtain basic labelling and length information, we converted molecules > 20 kb into bnx files with AutoDetect (BNG) software. We then aligned molecules > 180 kb that passed the quality control against each other, clustered them, and assembled them into contigs using the BNG assembly pipeline^53,54^. We used the *p-*values 1 × 10^–8^, 1 × 10^–9^, and 1 × 10^–15^ as thresholds for pairwise assembly, extension/refinement, and final contig refinement, respectively. We manually checked the resulting optical maps for chimeric contigs and resolved them when detected.

### Optical map length comparisons

Optical maps of Serr and 31.01 consisted of diploid and haploid regions. We self-aligned optical maps to identify diploid regions, decided which of the two haplotypes in the phased regions were present on the MS1-56 optical map, and disregarded the alternative haplotype. The sum of the edited Serr and 31.01 optical map lengths was compared with the length of the MS1-56 optical map.

### Pacific biosciences and 10X genomics sequencing

We isolated nuclear DNAs from MS1-56 as described earlier^55^ and prepared sequencing libraries using protocols from Pacific Biosciences (Menlo Park CA USA, henceforth). They were sequenced with the Single Molecule Real-Time (SMRT) technology at the Genome Center, University of California, Davis on the PacBio long-read sequencing instrument RSII using the P6 Polymerase Binding and C4 Chemistry Kits. Sequencing of the 58 PacBio SMRT cells of MS1-56 generated 57.2 Gb (~54X coverage) of reads averaging 12,791 bp with a maximum length of 82,702 bp. We assembled 2.05 million error-corrected qualified reads with Falcon (v0.4.2)^56^ into 460 contigs (total length of 1,056,053,412 bp), which we polished with the genomic consensus tool Quiver (https://github.com/PacificBiosciences/GenomicConsensus). We named this assembly MS1-56_v0.

To prepare 10X Genomics sequencing library, we isolated DNA from leaves of orchard-grown MS1-56. We prepared a sequencing library according to a protocol from 10X Genomics (Pleasanton CA USA). We sequenced the library with Illumina HiSeq4000 and assembled 680.53 million reads (89X genome equivalent, Table S1) with the 10X Genomics software Supernova (v1.1.3)^57^ using default parameters. The assembly produced 79,926 contigs with a total length of 1,005,111,912 bp and an N50 of 41,627 bp.

### Scaffolding

We digested 460 MS1-56 sequence contigs in assembly MS1-56_v0 in silico with the Nt.*Bsp*QI and Nb.*Bss*SI restriction endonucleases using Knickers (BNG) and aligned the contigs on the MS-156 Nt.*Bsp*QI and Nb.*Bss*SI optical maps using RefAligner (BNG). We visually checked the alignments in IrysView (BNG). Of the 460 MS1-56 polished contigs, 239 (~1047 Mb) were of sufficient length to be aligned. Five of the aligned contigs were chimeric and we disjoined them, increasing the total number of polished PacBio contigs to 465 (assembly MS1-56_v1, Table S2). The contig N50 = 7,904,778 bp. We then aligned MS1-56_v1 contigs on optical maps and scaffolded them with the hybrid-scaffolding pipeline (BNG). Since two optical maps were generated for MS1-56, we performed a two-step hybrid-scaffolding resolution with the strictest ‘conflict-free’ setting, first for sequence contigs aligned on the Nt.*Bsp*QI optical map. We then aligned scaffolds on the Nb.*Bss*SI optical map and scaffolded them further. We performed scaffolding also in the reverse order, using the Nb.*Bss*SI optical map for aligning sequence contigs, which was then followed by scaffolding on the Nt.*Bsp*QI optical map. We found no difference between the two approaches. BNG (https://bionanogenomics.com/support/software-downloads/) provided us with software packages for scaffolding.

To stitch the scaffolds, we trained the filtering parameters of Stitch^58^ to be suitable for the MS1-56 genome and then performed Stitch in iterations until we could produce no additional scaffolds. After each round of Stitch, we manually checked the ‘potentially chimeric scaffolds’ flagged by the program and resolved them if necessary. This sequential use of Stitch reduced the number of scaffolds from 465 to 264. The total length of this assembly (MS1-56_scf) was 1,066,408,726 bp, N50 = 34,776,948 bp, and 0.97% Ns.

### Phasing MS1-56 scaffolds into parental genomes

We aligned the 264 scaffolds of the MS1-56_scf assembly on the Serr and 31.01 optical maps using RefAligner (BNG). Of the 1,066,408,726 bp of the MS1-56 sequence, we allocated 536,956,484,537 bp present in 19 scaffolds to Serr and 527,846,219 bp, present in 21 scaffolds, to 31.01. This accounted for 99.85% of the assembly. The remaining 224 MS1-56 scaffolds had a total length of 1,606,023 bp (0.15% of the assembly) and were too short to be aligned on optical maps. We allocated those that could be aligned on the sequence of Chandler^11^ (88 scaffolds) and the remainder (136) we allocated to 31.01 by default. Thus, we allocated all 264 scaffolds to one of the two parental genomes: 107 to the Serr genome (JrSerr_v0.1), totaling 538,152,081 bp, and 157 to the 31.01 genome (Jm31.01_v0.1) totaling 528,256,645 bp.

### Pseudomolecule construction, gap closing, and error corrections

Eighteen Serr scaffolds equivalent to 532,266,604 bp and representing 98.9% of the Serr assembly and 19 of the 31.01 scaffolds equivalent to 523,690,948 bp and representing 99.1% of the 31.01 assembly shared SNP markers with the *J. regia* genetic and physical maps^17^ and a *J. microcarpa* genotyping-by-sequencing-produced map. We used these SNPs to ordered and orient scaffolds on the maps to construct pseudomolecules. The remaining 89 scaffolds (5,885,477 bp) of Serr and 138 scaffolds (4,565,697 bp) of 31.01 were short and could not be included into the pseudomolecules. There were two gaps of unknown size in the JrSerr_v0.1 pseudomolecules and three such gaps in the Jm31.01_v0.1 pseudomolecules, which we filled with 1,000 Ns. We estimated the lengths of the remaining 102 gaps in the JrSerr_v0.1 pseudomolecules and 106 in the Jm31.01_v0.1 pseudomolecules based on sequence alignments on the optical maps. The numbers and lengths of these gaps were reduced with unassigned scaffolds or sequence contigs assembled from 10X Genomics reads to 54 in both pseudomolecule sets with software FGAP v1.8.1^59^.

To correct errors remaining in the pseudomolecule sequences, we mapped about 93.9 Gb of Illumina reads (89 × ) onto the JrSerr_v0.1 and Jm31.01_v0.1 pseudomolecules using BWA v0.7.5^60^. We removed the duplicate reads present among the Illumina reads generated by PCR with SAMtools 0.1.19^61^. We performed single sample variant calling with SAMtools and BCFtools 0.1.19^62^ and kept variants with read depth > 10 and <200 and genotype quality > 30 and discarded the rest. We also discarded heterozygous read stacks. We aligned Illumina reads on the pseudomolecule sequences and corrected base substitutions and indels in the pseudomolecules with VCFtools^63^, which generated the final version of pseudomolecules and unanchored scaffolds JrSerr_v1.0 and Jm31.01_v1.0.

### TE annotation and SSR discovery

We identified repeated sequences in the JrSerr_v1.0 and Jm31.01_v1.0 assemblies by a combination of homolog-based and *de novo* approaches. We used the software RepeatModeler v1.0.10 (http://www.repeatmasker.org/RepeatModeler) to obtain TE consensus sequences. We deployed MITE-Hunter^64^ to identify Miniature Inverted Repeat Transposable Elements (MITEs) and SINE-Finder^65^ to identify Short Interspersed Nuclear Elements (SINEs). We manually inspected the outputs to eliminate artifacts and combined the TEs which passed the quality control with the current release of Repbase v22.06^66^ and the *J. regia* repetitive library to produce a custom TE library. We used the custom library to predict TEs with software RepeatMasker v4.0.7 (http://www.repeatmasker.org). We classified the TE sequences according to the system proposed by Wicker et al.^67^.

We used the MISA^68^ software to identify microsatellite motifs in the assemblies with the following minimum repeat numbers: ten for monomers, six for the di-, and five for the tri-, tetra-, penta-, and hexanucleotide motifs. We used a 1-Mb sliding window to calculate the SSR density on each chromosome.

### Gene annotation and transcription factor and R gene identification

We used both homology-based and *de novo* methods to predict protein-coding genes in the TE masked JrSerr_v1.0 and Jm31.01_v1.0 assemblies. We extracted homologous protein sequences for eudicots from NCBI and used them as training sets in Augustus^69^. We used a *J. regia* gene set^11^ to create species-specific parameters for Augustus and used these parameters also in *de novo* gene prediction by Maker^70^. For homology-based gene annotation, we used the transcripts of *J. regia*^11^ to generate predicted homology-based gene models in Maker^70^. Finally, we merged the gene models from de novo prediction and homology-based prediction to produce a comprehensive and non-redundant gene set by using Maker^70^.

We used the Benchmarking Universal Single-Copy Orthologs (BUSCO) v3 tool^71^ to identify universal single copy orthologs (USCOs) in the assembly as a measure of completeness. BUSCO analysis was performed using the plant dataset (embryophyta_odb9), which excludes the *Physcomitrella patens* and *Selaginella moellendorffii* and contained 1440 BUSCO groups.

We used DNA-binding domains in Pfam 31.0^72^ to identify transcription factors (TFs) among the protein-coding genes annotated in JrSerr_v1.0 and Jm31.01_v1.0 using BLAST. We classified the TFs according to the Plant Transcription Factor Databases (plantTFDB, http://planttfdb.cbi.edu.cn/).

R genes were identified using the prediction pipeline RGAugury^73^. Amino acid sequences of the protein coding genes in the whole genomes and their coordinates on the genomes (gff3 files) were used as the input. An E value of 1 × 10^−5^ was used for the protein BLAST search against four protein databases: pfam, smart, gene3d, and superfamily. Four types of RGAs were predicted, including nucleotide‐binding sites encoding genes, RLK, receptor‐like protein, and transmembrane coiled‐coil protein through screening for the presence of major domains, such as NB‐ARC, toll‐interleukin receptor, leucine‐rich repeats, coiled‐coil domain, transmembrane domain, serine/threonine kinase domain, and LysM motif.

To determine if the distribution of the R genes along chromosomes mirrored that of other genes, the total number of genes along a chromosome arm were divided into 20 windows, each containing the same number of genes. The proportion of R genes in each window was computed and averaged across the 32 chromosome arms. Rank correlation coefficients (*r*) for the window location on the telomere-to-centromere axis and the mean proportion of R genes in the window were computed for both genome assemblies.

### Dot-plots and circular plots

To construct synteny dot-plots, we downloaded protein sequences for *Vitis vinifera* (Genoscope)^18^ and *Amborella trichopoda* v1.0^19^ from Phytozome v12.0 and used them in BLASTP searches as targets while genes from JrSerr_v1.0 were used as queries. We also aligned JrSerr_v1.0 and Jm31.01_v1.0, in which we used the former as a query source and the latter as a target in BLASTP with an E value of 1 × 10^−5^. We analyzed all alignments with MCScanX^74^ with default parameters. We also use MCScanX with default parameters to find collinear blocks containing at least five collinear gene pairs. We visualized syntenic blocks in Circos 0.69^75^ and constructed the dot-plots with dot_plotter in MCScanX^74^.

To identify WGD within *J. regia*, we searched homology by BLASTP using HC genes annotated on the JrSerr_v1.0 pseudomolecules both as queries and targets (self-search). We performed the same analysis with HC genes annotated on the Jm31.01_v1.0 pseudomolecules. We used a filter threshold of 1 × 10^–5^ to identify homologous proteins. The longest protein sequences were analyzed with MCScanX with default parameters. We considered at least five genes in an ascending or descending order as a syntenic block. We constructed the syntenic block maps with Circos 0.69 software^75^ and dot-plots with dot_plotter in MCScanX^74^.

### Molecular clock rate

We used the JrSerr_v1.0, Jm31.01_v1.0, *V. vinifera*, and *A. trichopoda* gene sequences to calculate the synonymous (*Ks*) substitution rates. We identified orthologous genes among these four species using Proteinortho5^76^. We identified orthologous inter-genome gene pairs and paralogous intra-genome gene pairs by bi-directional BLAST with the default parameter. We aligned amino-acid sequences of each pair of genes using ClustalW^77^ and transformed amino-acid alignments into nucleotide sequence alignments according to coding sequences (CDS). We then calculated the *Ka* and *Ks* of each pair of genes using Kimura 2 parameter model in KaKs_Calculator2.0^78^. We computed genome divergence times and dated the Juglandoid WGD or γWGT according to *r* = *Ks*/2*t*, where *r* is the rate of nucleotide substitution per synonymous site per year, *Ks* is the number of substitutions per synonymous site, and *t* is time of species divergence in years. We used the same approach to compute *r* from *Ka*. Using the time estimate and the average *Ka* and *Ks* between paralogues on the homoeologous chromosomes in the Serr and 31.01 genomes, we computed clock rates.

### Gene collinearity and structural chromosome analyses

The methodology used for gene collinearity analysis has been described earlier^14^, and we will provide only essential information. We used the amino-acid sequences of 31,286 HC genes annotated in the JrSerr_v1.0 pseudomolecules as a query and the amino acid sequences of grape HC genes as targets in BLASTP homology searches. We used a default BLASTP parameter setting. We also performed BLSTP search using JrSerr_v1.0 as a query against itself (target). We recorded the top three alignments. In the BLASTP searches using JrSerr_v1.0 as a query against Jm31.01_v1.0 as a target, we recorded only the top hit.

We analyzed collinearity as follows. We considered three or more genes to be collinear if the starting nucleotides of the top hits followed an ascending or descending order and distances between them were <0.5 Mb, allowing non-collinear genes to interrupt a sequence of collinear genes. If a gene was homologous to tandem duplicated genes on a target pseudomolecule, we recorded only one of the duplicated genes as collinear, provided that it was in a collinear position on the pseudomolecule.

We constructed a spreadsheet of datasets showing collinearity of each of the 31,286 genes on the JrSerr_v1.0 pseudomolecules with those on the JrSerr_v1.0 pseudomolecules (self), with those on the Jm31.01_v1.0 pseudomolecules, and the grape pseudomolecules. We colored spreadsheet cells containing collinear genes whereas those that were not collinear we left colorless. We indicated changes in gene order due to inversions or translocations by changes in cell color. We recorded for each rearrangement the register of the starting and ending gene and rearrangement type. We coded the rearrangements as follows. A = inversion of 2 genes, B = inversion of 3 genes, C = inversion of > 3 genes, D = translocation of 2 genes within a chromosome, E = translocation of 3 genes within a chromosome, F = translocation > 3 genes within a chromosome, iT = interstitial translocation between chromosomes, T = terminal translocation, Dup = duplication of a segment, and Del = deletion of a segment. To quantify collinearity, we counted genes of JrSerr_v1.0 collinear with a specific genome and expressed them as a percentage of all HC genes present on the JrSerr_v1.0 pseudomolecules.

We compared the order of collinear genes along pseudomolecules within JrSerr_v1.0, between JrSerr_v1.0 and Jm31.01_v1.0, and between JrSerr_v1.0 and the grape pseudomolecules and determined the most parsimonious origin of a rearrangement, provided that information on the rearrangement was available in all genomes and subgenomes. We coded the branch assignments as follows: *J. microcarpa* (M), *J. regia* (R), and the branch preceding the divergence of the two (RM). We used only major rearrangements (C, F, and iT) in computing rates. We computed the rearrangement rate acquisition by a branch using time estimates in Table S13.

We used the following approach to quantify gene collinearity between the JrSerr_v1.0 pseudomolecules and the grape pseudomolecules. We recorded only the top hits in an effort to identify the orthologous pseudomolecule among the three homoeologous pseudomolecules in the grape genome. Since this approach still produced multiple alignments for every JrSerr_v1.0 pseudomolecule in the grape genome, we selected the alignment that contained the largest number of collinear genes and eliminated the remaining alignments present. We color coded the collinear genes on the orthologous alignments and counted them.

### Fractionation of the Juglandoid WGD

To investigate the distribution of the differences in gene numbers along the dominant and subdominant chromosomes, we subdivided each homoeologous chromosome pair into *N* intervals between *N* + 1 collinear genes shared by the homoeologous chromosome pairs in the Serr genome. We then counted the number of unique genes (henceforth singletons) present in an interval and plotted the numbers for each homoeologue. We tested the significance of the difference in singleton numbers between the dominant and subdominant chromosomes with the paired *t*-test using the numbers in individual intervals in the dominant and subdominant chromosomes as variables.

To express the number of singletons per Mb rather than per interval between collinear genes, we subdivided the dominant chromosome into 2-Mb intervals and counted and plotted the number of singleton genes in the 2-Mb interval.

### Gene expression between dominant and subdominant chromosomes of *J. regia*

We downloaded the expression data for 22 RNAseq libraries^11^ and measured dominance in gene expression between pairs of collinear orthologues located on the eight homoeologous chromosome pairs of *J. regia*. We quantified gene expression level using FPKM values in Cufflinks^79^. We considered collinear orthologous pairs with greater than two-fold difference^80^ as differentially expressed. We termed the gene that showed higher expression in differentially expressed gene pairs as the dominant orthologue whereas the gene with lower expression as the subordinate collinear orthologue. We counted the number of dominant genes in each chromosome and performed paired *t*-test to compare the difference in the number of dominant genes between dominant and subdominant subgenomes.

### Phylogeny reconstruction

We downloaded the following genome assemblies for phylogenetic analysis and the construction of divergence chronology: *Vitis vinifera*^18^, *Prunus persica*^81^, *Amborella trichopoda*^19^, *Populus trichocarpa*^35^, *Theobroma cacao*^82^, *Malus domestica*^83^, and *J. regia* Chandler, *J. sigillata*, *J. nigra*, *J. microcarpa* acc. 29.01, *J. hindsii*, *J. cathayensis*, *Pterocarya stenoptera*^5^. With Proteinortho^76^ using default parameters we identified 1155 single-copy, orthologous genes shared by the *J. regia* (JrSerr_v1.0), *J. microcarpa* (Jm3101_v1.0), *Amborella trichopoda*, *Vitis vinifera*, *Malus domestica*, *Theobroma cacao*, *Prunus persica*, and *Populus trichocarpa* genomes. We aligned amino-acid sequences of the 1,155 genes in the eight genomes with MAFFT^84^, removed regions with gaps, and concatenated the remaining sequences. We used the phylogenetic software IQ-TREE^85^ employing the Maximum-likelihood method to infer the phylogeny of the eight genomes and performed 1000 bootstrap replications to produce a consensus tree. We constructed the final phylogenetic tree with Figtree^86^.

Because neither pseudomolecules nor gene annotation was reported for *J. regia* Chandler, *J. sigillata*, *J. nigra*, *J. microcarpa* acc. 29.01, *J. hindsii*, *J. cathayensis*, and *Pterocarya stenoptera*^5^, we aligned the CDS sequences of the 1,155 Serr and 31.01 genes to these seven genomic sequences to obtain corresponding CDS sequences. We then constructed a phylogenetic tree for the fifteen genomes as described above with the following changes. We converted the CDS sequences to amino-acid sequences. After removing partial alignments, all 15 genomes shared 809 single-copy, orthologous genes. We aligned the gene sequences with MAFFT^84^, removed regions with gaps, concatenated the remaining sequences, and constructed a tree.

### Divergence time estimation

Based on fossil record, the diversification of Juglandaceae was estimated to have been initiated 80 MYA (71–96 MYA 95% CI)^24^. Since this estimate includes speciation of diploid *Rhoiptelea*, the WGD must have occurred after this date. Based on the chronogram in^24^ and fossil dating of the Engelhardioideae and Juglandoideae divergence to upper Cretaceous^21,87^ we arbitrarily chose 66 MYA for the origin of the Juglandoid WGD (reviewed in^88^). Based on the non-synonymous (*Ka*) and synonymous (*Ks*) divergence values computed for 1,429 collinear genes on the Serr homoeologous chromosome with KaKs Calculator 2.0^78^, we calibrated the molecular clock. We used MACSE^89^ to perform multiple sequence alignment for each gene using the method of model averaging. The divergence (*k*) of pairs of orthologous genes was converted into time of divergence (*t*) in million years using formula: *t* = *k*/(2*r*)/10^6^.

## Supplementary information

Supplemental Information

File S1

File S2

File S3

File S4

File S5

File S6

## Data Availability

The genome assemblies and gene annotations of *J. regia* Serr and *J. microcarpa* acc. 31.01, and all sequencing data have been deposited under NCBI BioProject PRJNA413991. The assemblies, TE and gene annotations of *J. regia* Serr and *J. microcarpa* acc. 31.01 are also accessible via the JBroswer at http://aegilops.wheat.ucdavis.edu/jbrowse/index.html?data = JrSerr&loc and http://aegilops.wheat.ucdavis.edu/jbrowse/index.html?data = Jm31.01&loc.
