# Supplementary material for: Sequencing a Juglans regia × J. microcarpa hybrid yields high-quality genome assemblies of parental species
Source: Hortic Res. 2019 Mar 25;6:55. doi: 10.1038/s41438-019-0139-1 (PMC6431679; doi:10.1038/s41438-019-0139-1)
Supplement: Supplementary file 1 — Supplemental Information [file 41438_2019_139_MOESM1_ESM.docx]

**Supporting Information**

| 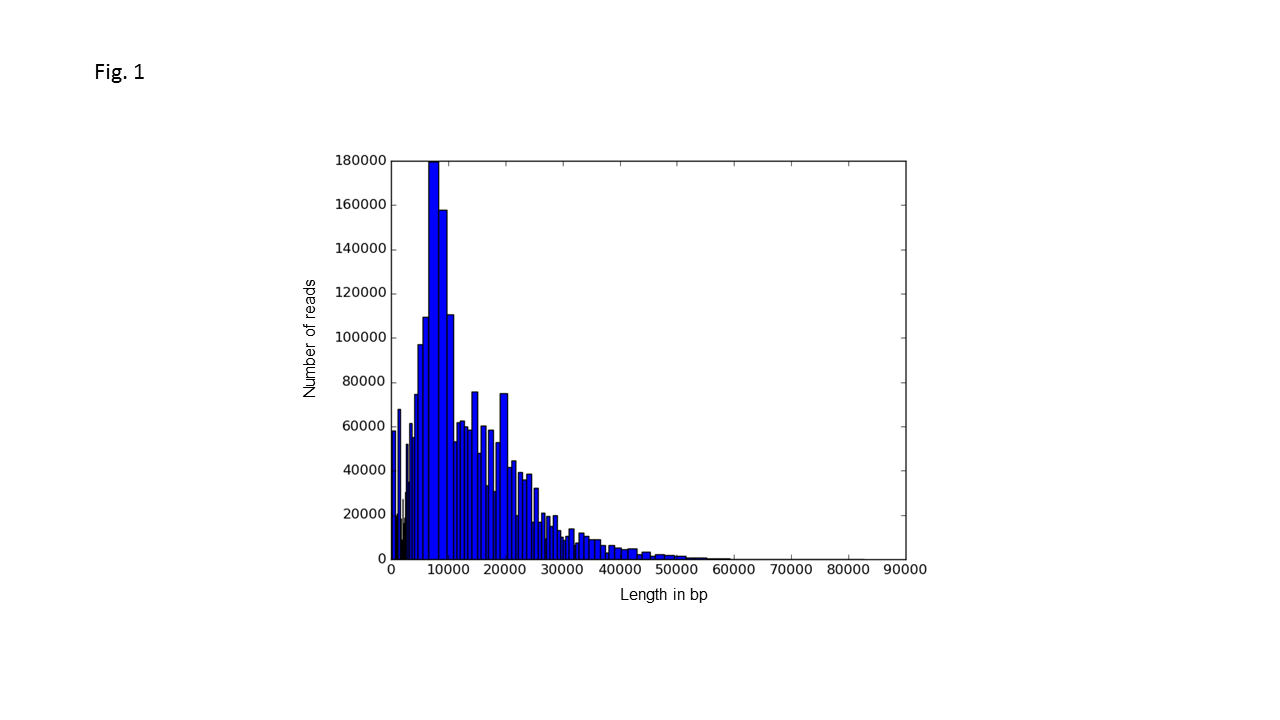 |
| --- |
| **Fig. S1. Pacific Biosciences WGS reads**. Numbers of reads (vertical axis) of indicated lengths produced by sequencing 58 SMRT cells of hybrid MS1-56 (horizontal axis). |

| **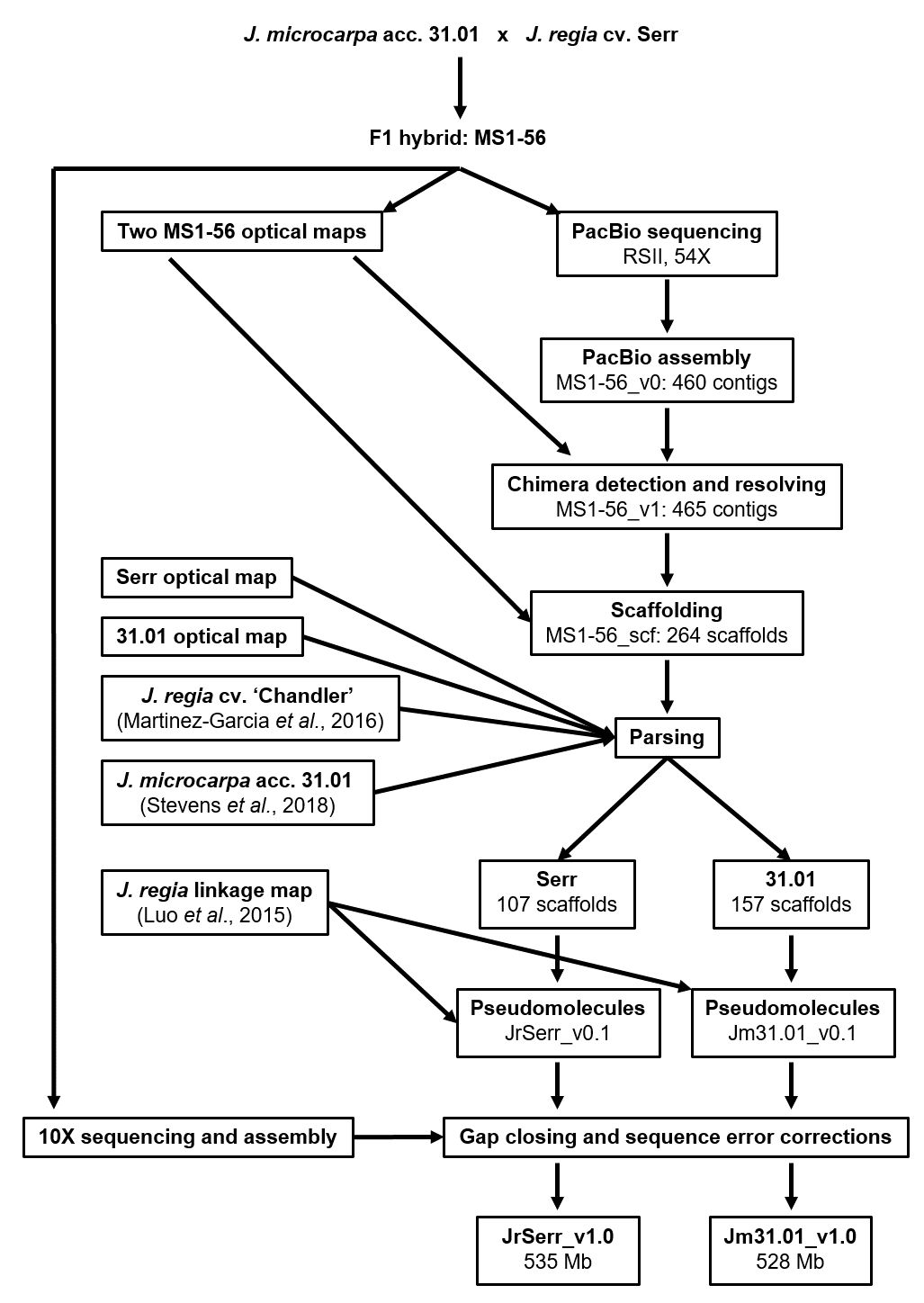** |
| --- |
| **Fig. S2. Sequence assembly flowchart.** MS1-56 hybrid sequence assembly, scaffolding, scaffold allocation to parental genomes, pseudomolecule construction, and gap closing. |

| **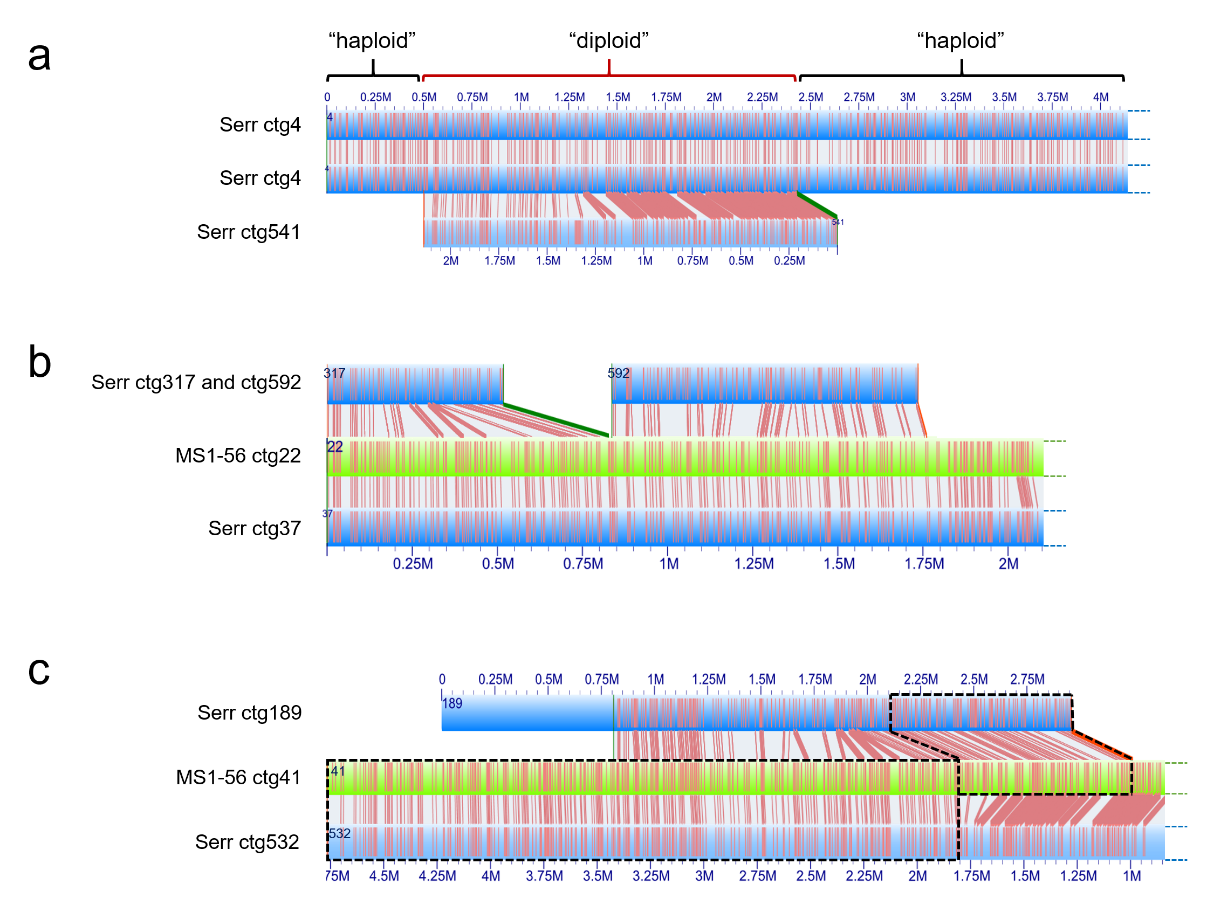** |
| --- |
| **Fig. S3.** **Optical map self-alignments and haplotype phasing** (a) Phased contigs in self-aligned Serr optical map showing two “haploid” regions in contig ctg4**,** in which the two Serr haplotypes were collapsed, and a “diploid” region, in which the Serr haplotypes were phased into separated contigs (ctg4 and ctg541) during optical map assembly. (b,c) Alignments of phased Serr haplotypes (blue) on the optical map of the MS1-56 hybrid (green) and identification of haplotypes that were transmitted from Serr to MS1-56 for editing of the Serr optical map. In b, Serr haplotype in contig ctg37 was transmitted to contig MS1-56 ctg22. Redundant homologous haplotypes in contigs Serr ctg317 and 592 were disregarded during editing of the Serr optical map. In c**,** a Serr recombined haplotype of contigs ctg532 and ctg189 was transmitted to contig MS1-56 ctg41. Redundant portions of Serr haplotypes in contigs ctg189 and ctg532 were disregarded during editing of the Serr optical map, as shown. |

| 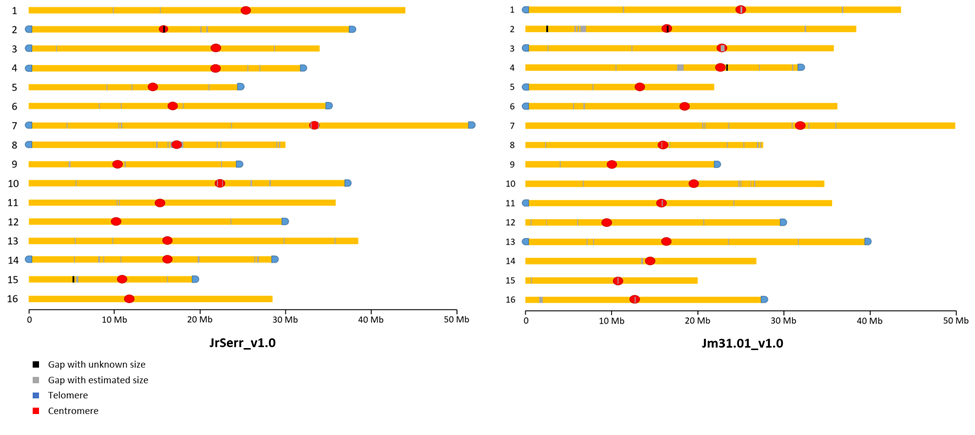 |
| --- |
| **Fig. S4**. **Ideograms of *Juglans regia* Serr (JrSerr_v1.0) and *J. microcarpa* 31.01 (Jm31.01_v1.0) pseudomolecules**. The gray bars indicate the locations of gaps of lengths estimated with the aid of the optical map and the black bars indicate the locations of gaps of unknown lengths. The locations of centromeres and sequenced telomeres are indicated. |


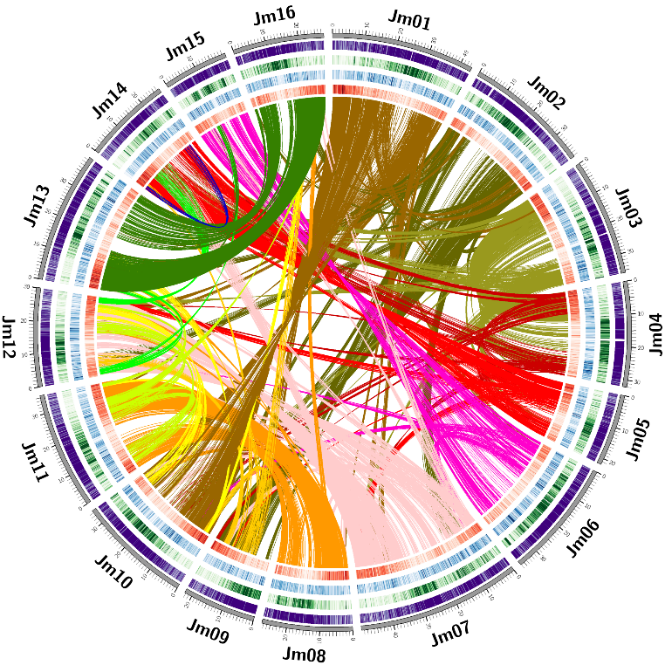


**Fig. S5.** **Circular plots of the *J*. *microcarpa* pseudomolecules**. Circles from outside to inside are: pseudomolecule coordinates in Mb, density of all types of LTR-RTs, density of *Gypsy* LTR-RTs, density of *Copia* LTR-RTs, and gene density. Central lines connect syntenic blocks across chromosomes. Different colors represent different homoeologous chromosome pairs derived from the Juglandoid WGD.

| 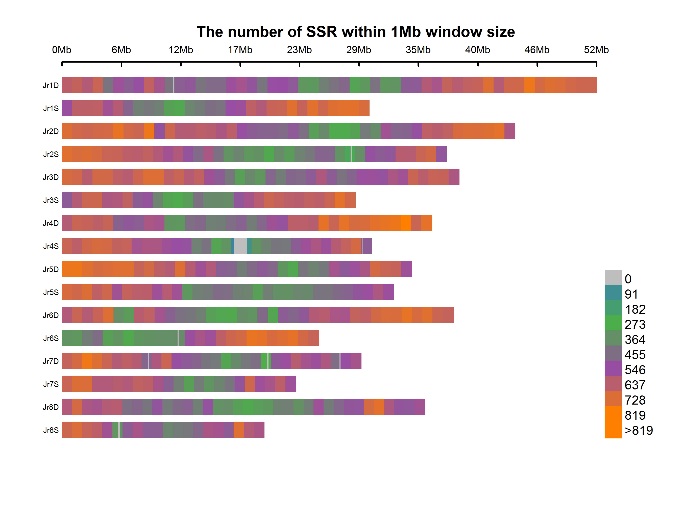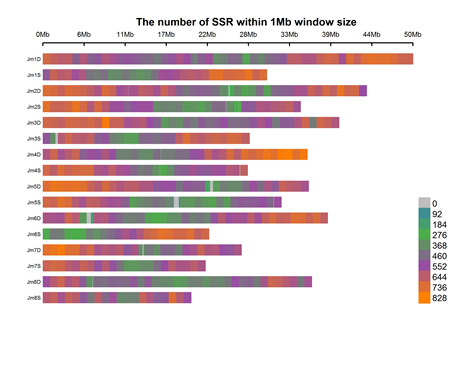 |
| --- |
| **Fig. S6. SSR density along *Juglans regia* and *J. microcarpa* chromosomes**. Heat maps show the numbers of SSRs per 1Mb nonoverlapping window along the JrSerr_v1.0 pseudomolecules (left panel) and along the Jm3101_v1.0 pseudomolecules (right panel). The starting nucleotides of the pseudomolecules are to the left. |
| 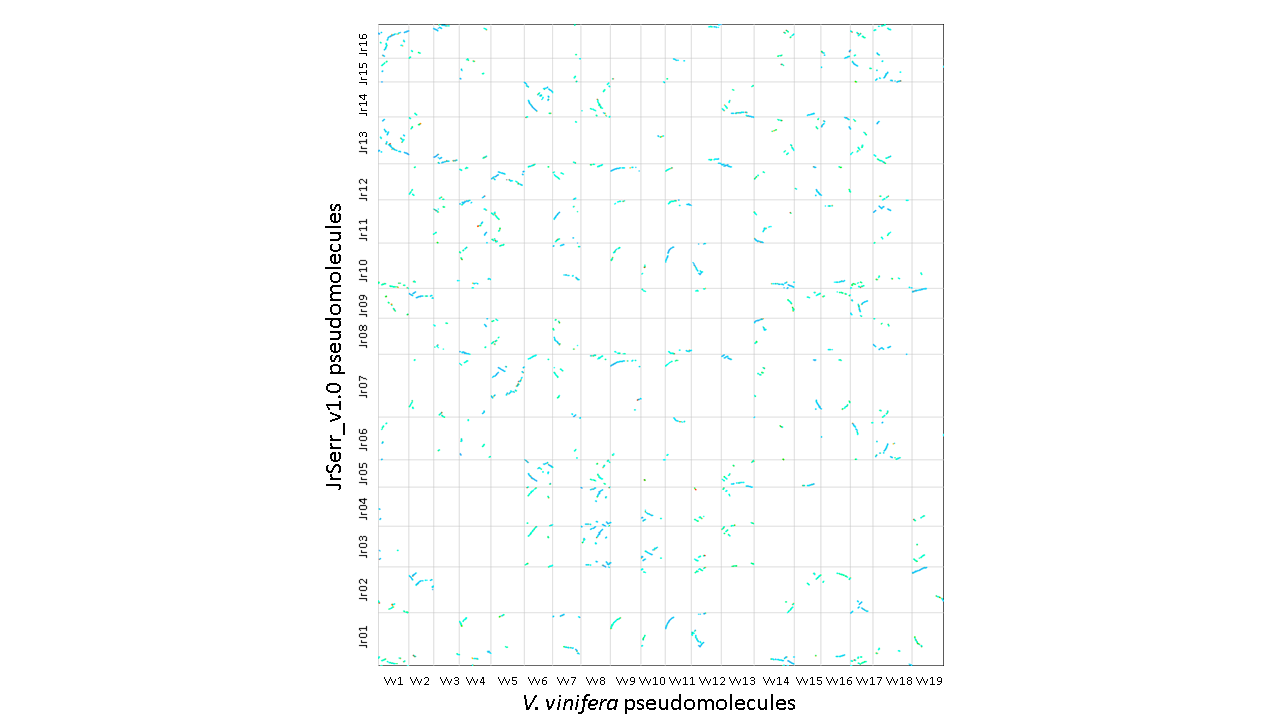 |
| **Fig. S7**. **Synteny of *Juglans regia* (JrSerr_v1.0) pseudomolecules with those of grape (*Vitis vinifera*)**. The starting nucleotides of the *J. regia* and *V. vinifera* pseudomolecules are to the left (grape) and bottom (*J. regia*). In many cases, a single synteny block in *V. vinifera* is homoeologous to two synteny blocks in *J. regia* due to the Juglandoid WGD. Some *J. regia* regions are homoeologous to two or three synteny blocks in *V. vinifera* due to the γWGT. |

| 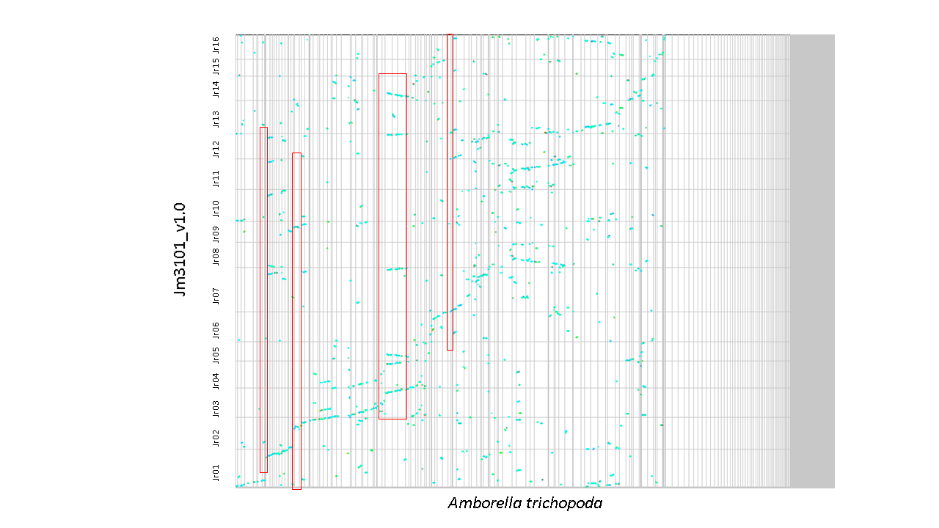 |  |
| --- | --- |
| **Fig. S8**. **Synteny between *Juglans regia* (JrSerr_v1.0) pseudomolecules and *Amborella trichopoda* scaffolds**. Starting nucleotides of *J. regia* pseudomolecules are at the bottom. Some single *A. trichopoda* scaffolds (horizontal axis) are homoeologous to six synteny blocks in *J. regia* Serr (boxed in red). However, no *J. regia* pseudomolecule appears to be homoeologous to more than one *A. trichopoda* scaffold. This pattern is due to the γWGT and the Juglandoid WGD, which both occurred after the divergence of the *J. regia* and *Amborella* lineages. |  |
| 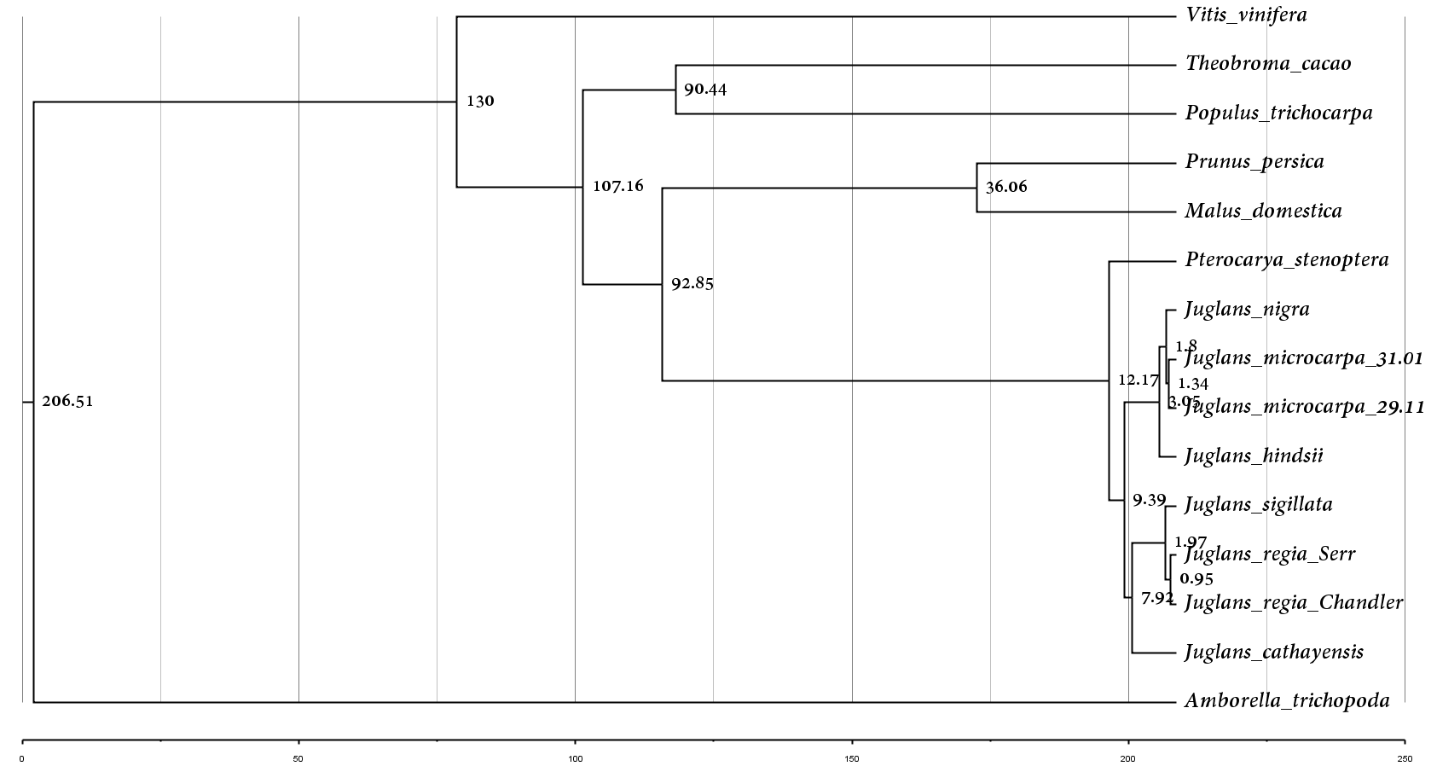 | |
| **Fig. S9**. **Chronogram of 15 genomes of woody perennials**. The phylogenetic tree was constructed from alignment of 809 orthologous, single-copy genes shared by the 15 genomes. Genome divergence times were estimated using MEGA X^1^ using a grape-poplar divergence time set at 110 MYA-130 MYA, which is similar to our grape-*Juglans* divergence time estimated in Table S13. Scale is in MY. | |

**
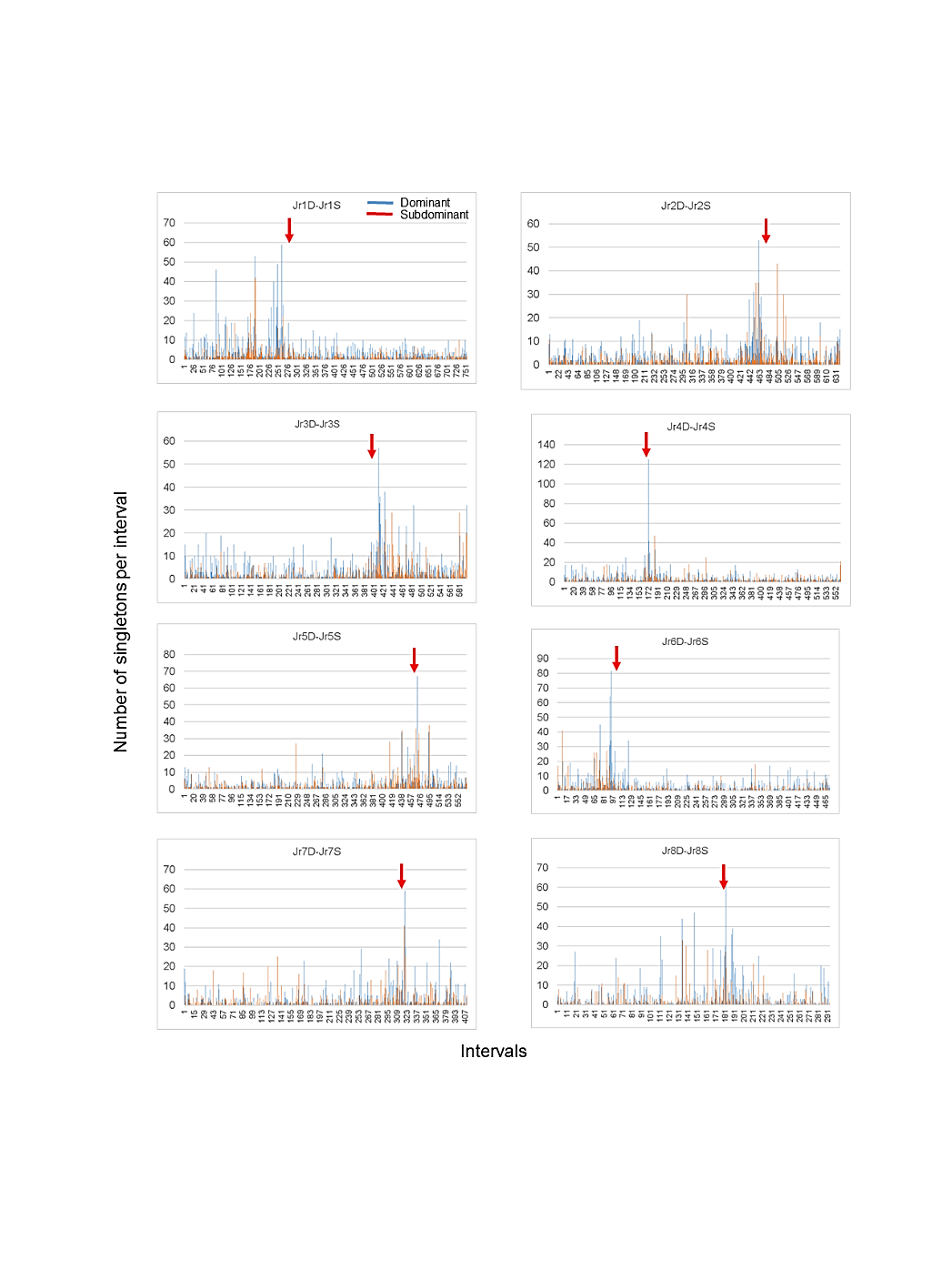
**

**Fig. S10.** **Asymmetric gene fractionation of the Juglandoid WGD**. Each pair of homoeologous pseudomolecules in the JrSerr_v1.0 genome sequence was subdivided into intervals delimited by successive pairs of paralogous genes in collinear locations on the homoeologues. The noncollinear genes (singletons) were counted in each interval (histogram bars) in the dominant pseudomolecule and homoeologous subdominant pseudomolecule. The starting nucleotides of the pseudomolecules are to the left. Arrows indicate the locations of the centromeres in the dominant pseudomolecules.

| 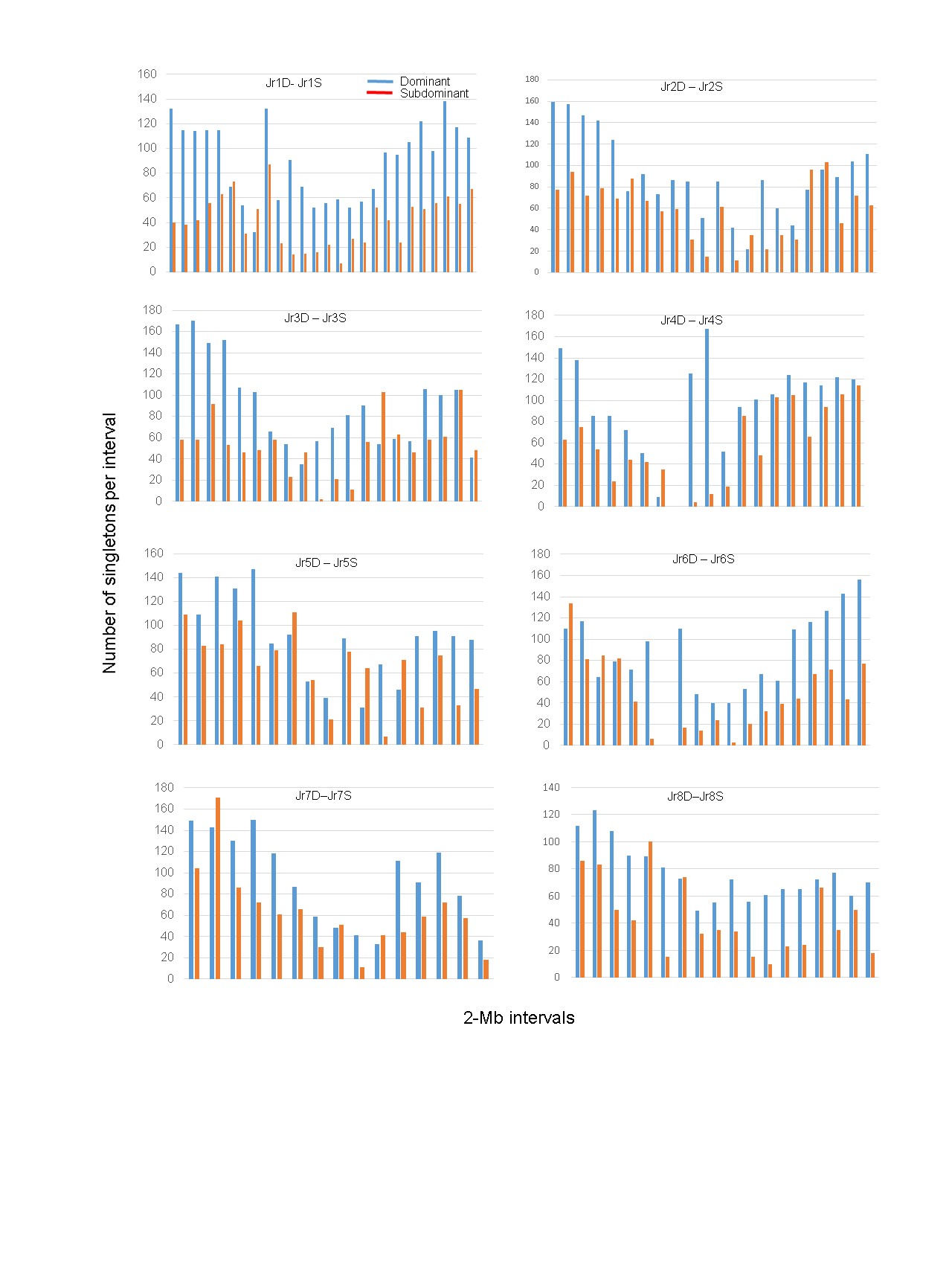 |
| --- |
| **Fig. S11. Gene loss from *Juglans regia* dominant and subdominant homoeologous pseudomolecules**. Each bar represents the number of singleton genes per 2-Mb non-overlapping window (the last window may be less than 2 Mb). Blue bars are the dominant chromosomes and the red bars are subdominant chromosomes. The starts of the pseudomolecules are to the left in each histogram. |

**
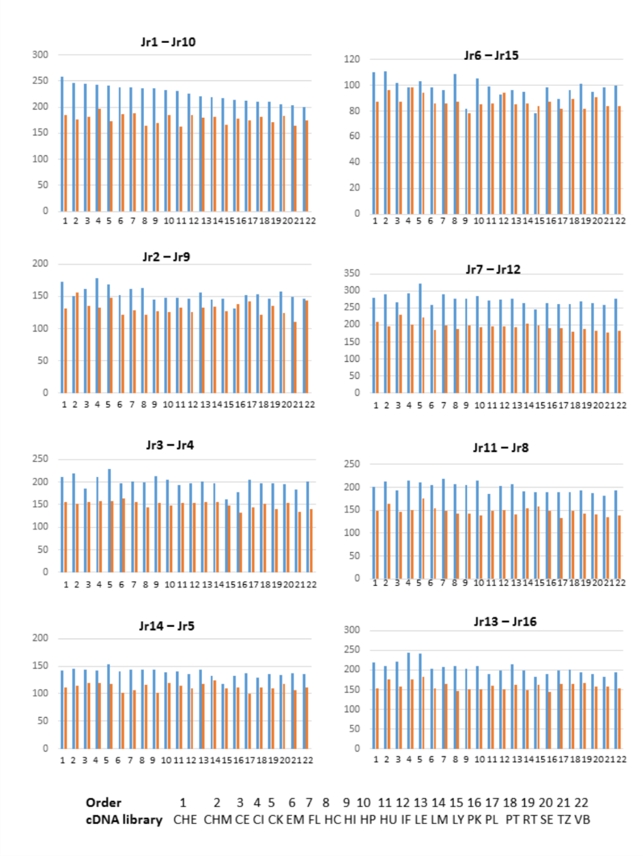
**

**Fig. S12.** **Numbers of genes on dominant (blue) and subdominant (red) homoeologous chromosomes with a dominant expression in 22 different *J. regia* RNAseq datasets**. We define dominant expression as two-fold difference in expression. The description of the RNAseq datasets (coded at the bottom of graphs) is in **Table S20.**

| 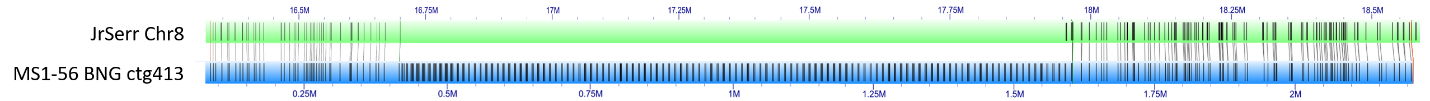 |
| --- |
| **Fig. S13. The array of the 18S-5.8S-26S rRNA gene units on *Juglans regia* chromosome Jr4S.** The pseudomolecule (green rectangle) is on top and optical map contig is on the bottom (blue rectangle). The pseudomolecule coordinates of the array are given in Mb above the pseudomolecule. The coordinates below the optical map contig are relevant only to the optical map. The black vertical lines connect corresponding restriction sites in the pseudomolecule and the optical contig. Note the periodicity of the restriction sites along the optical map contig which are present as a gap of estimated length in the pseudomolecule. Only a single 18S-5.8S-26S rRNA unit was present in the pseudomolecule. |
|  |
| **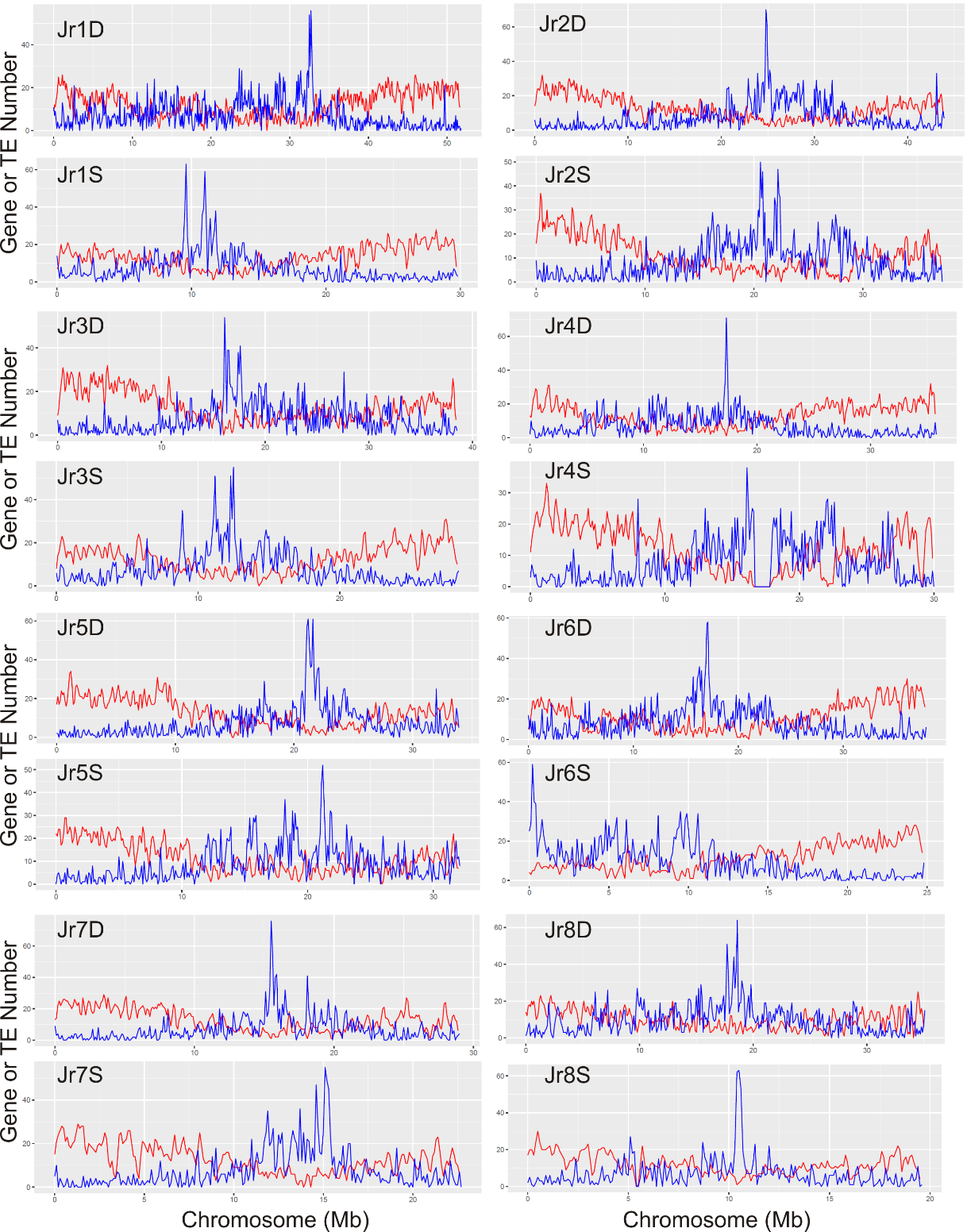**  **Fig. S14. The distribution of genes (red) and transposable elements (blue) along the 16 *Juglans regia* pseudomolecules**. A 1-Mb sliding window was used to compute the gene and TE densities along each pseudomolecule. The gene density declined gradually in the distal to proximal direction, while the TE density increased in the distal to proximal direction. |

| **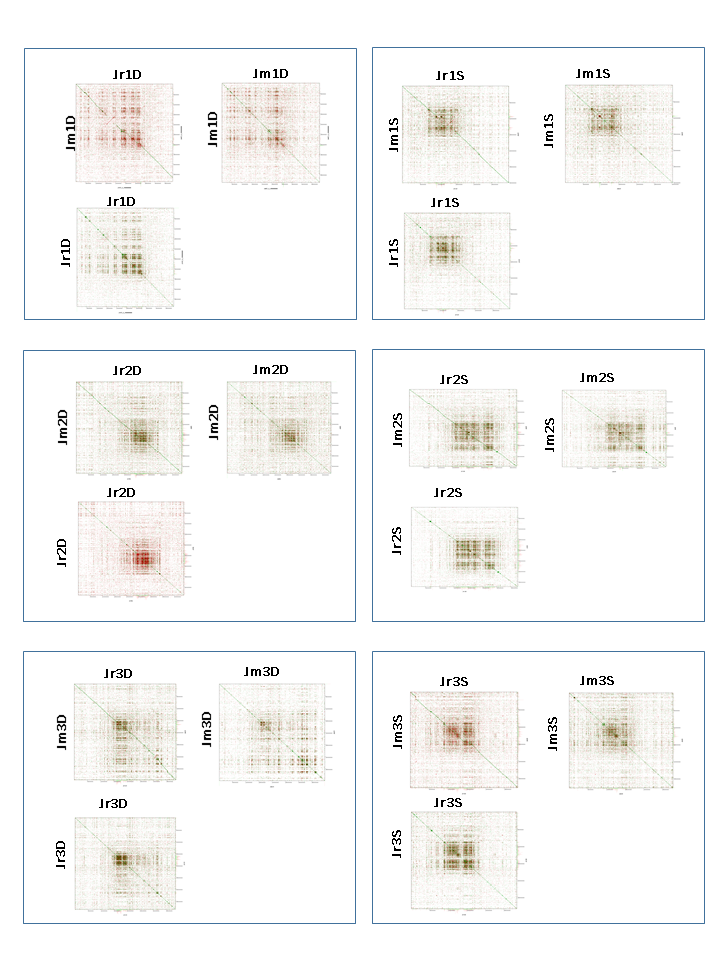** |
| --- |
| **Fig. S15**. **Dot-plot alignments of the JrSerr_v1.0 and Jm31.01_v1.0 pseudomolecules.** Shown are dot-plots of the JrSerr_v1.0 and Jm31.01_v1.0 self-aligned pseudomolecules and JrSerr_v1.0 aligned against Jm31.01_v1.0 pseudomolecules. The alignments show the locations of centromeric regions and provide evidence that the same repeated sequences are located in those regions in the corresponding *Juglans regia* and *J. microcarpa* chromosomes. |

| 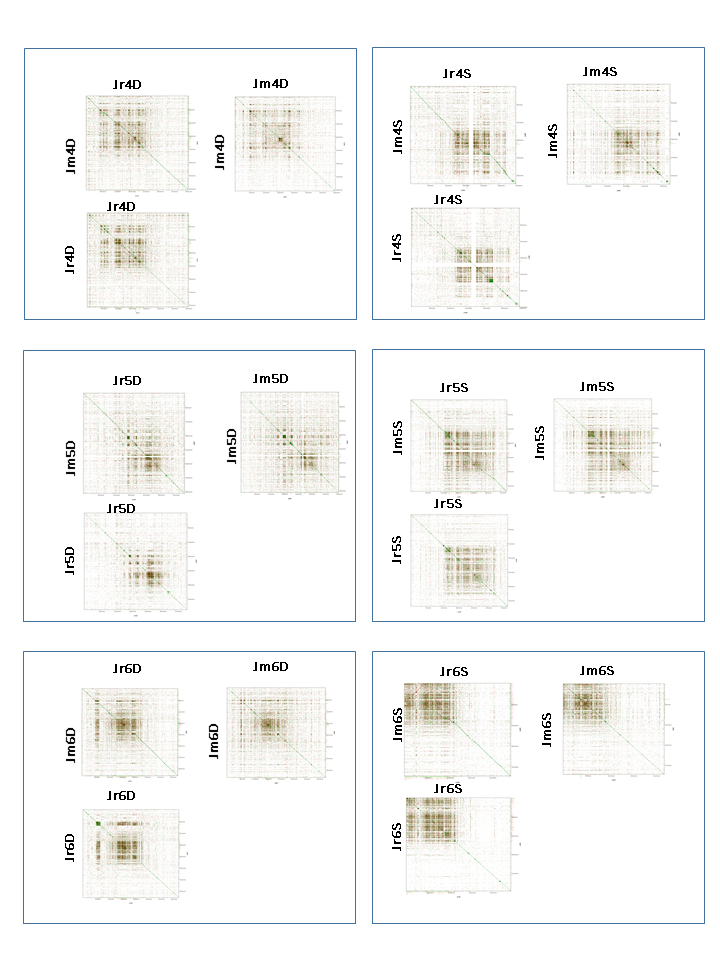 |
| --- |
| **Fig. S15** cont. |

| 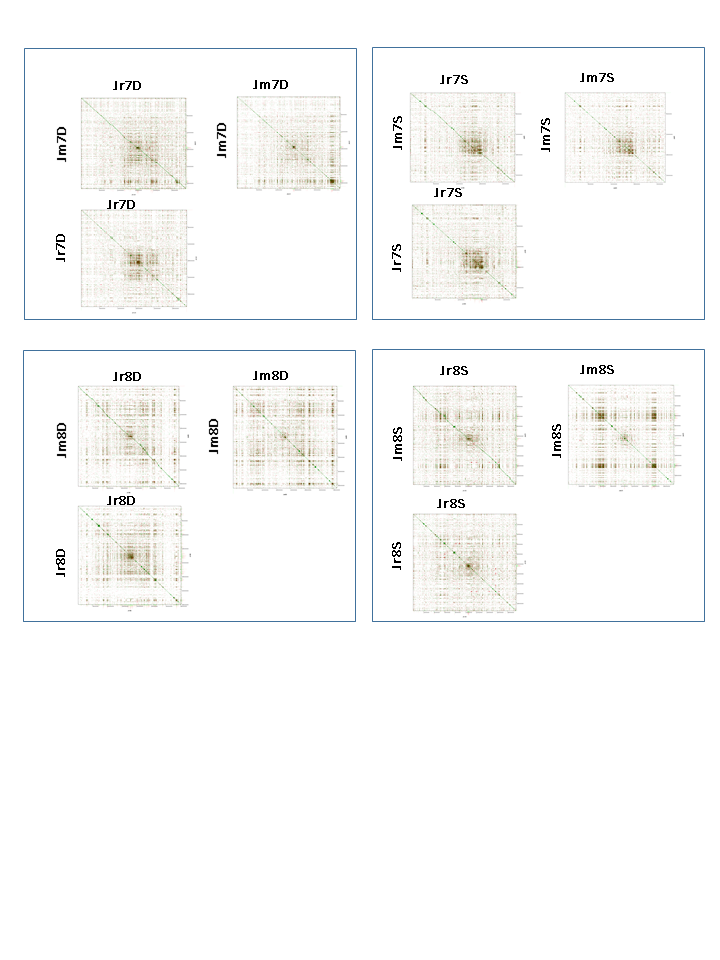 |
| --- |
| **Fig. S15** cont. |


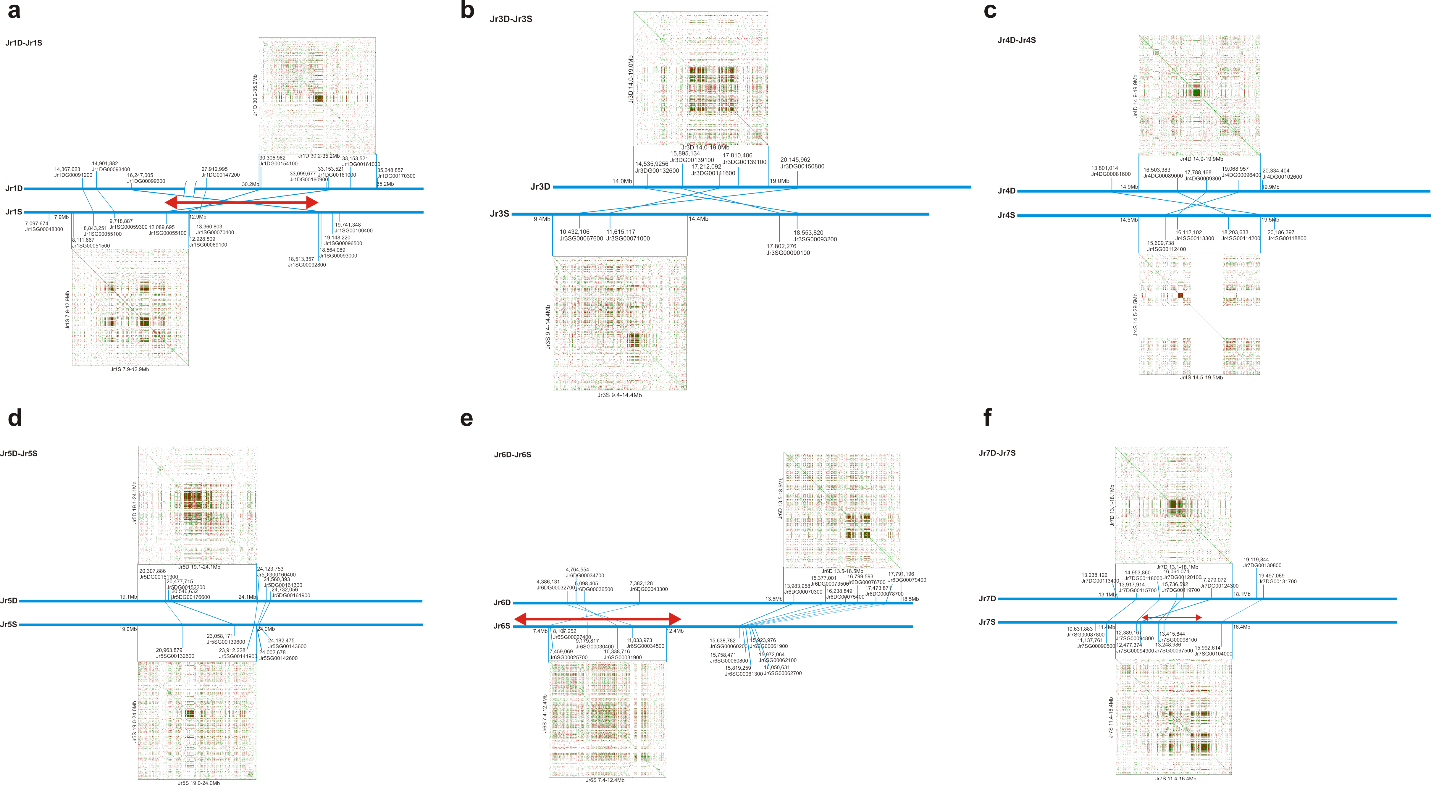
**Fig. S16. Alignment of centromeric regions on six pairs of homoeologous pseudomolecules in *Juglans regia*.** We employed selected collinear paralogous genes (connected by thin lines) located in the vicinity or within of a 5-Mb interval surrounding the *JCR* centromeric array on homoeologous pseudomolecules to align the dot-plots of the 5-Mb regions relative to each other. More such genes existed (**File S1**) but only some were used here. Each dot-plot is a self-alignment of a 5-Mb region surrounding the *JCR* array. Forward matches are in green and reverse matches are in red in the dot-plots. Inversions (red double-head arrows) relevant to repositioning of a *JCR* array are indicated. Note that pseudomolecule 3S is inverted relative to 3D and pseudomolecule 4S is inverted relative to 4D.

| 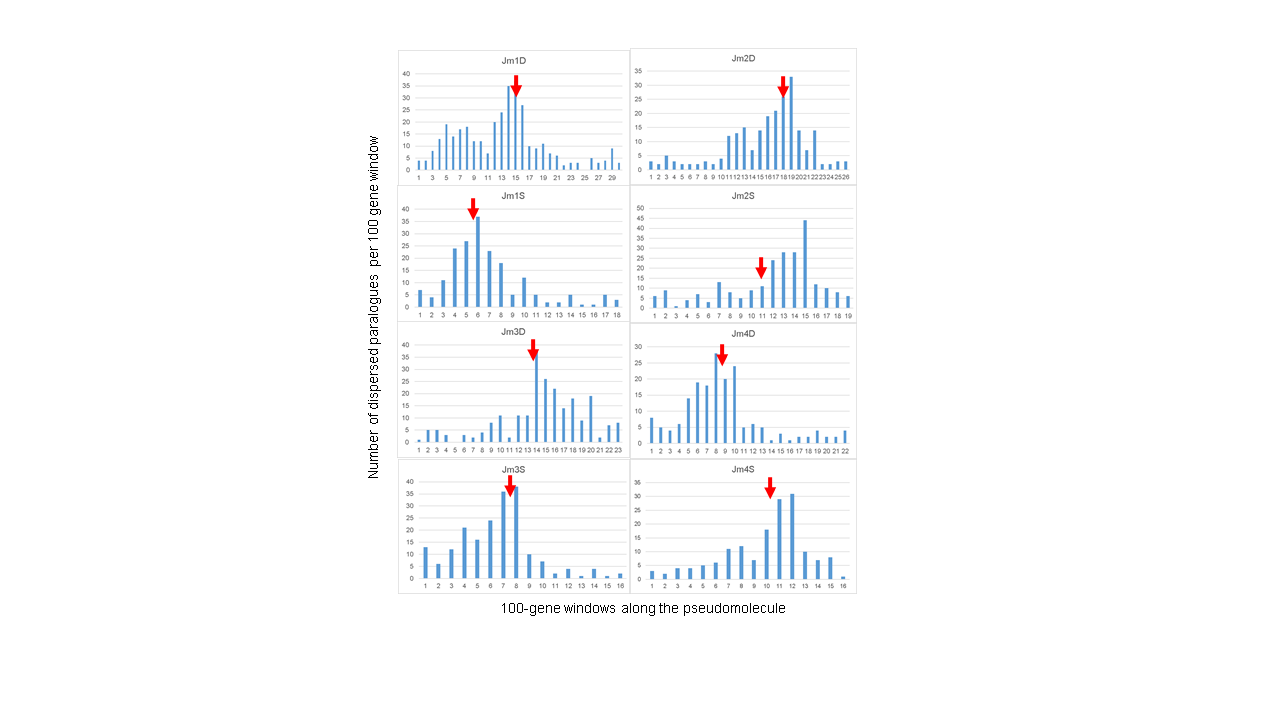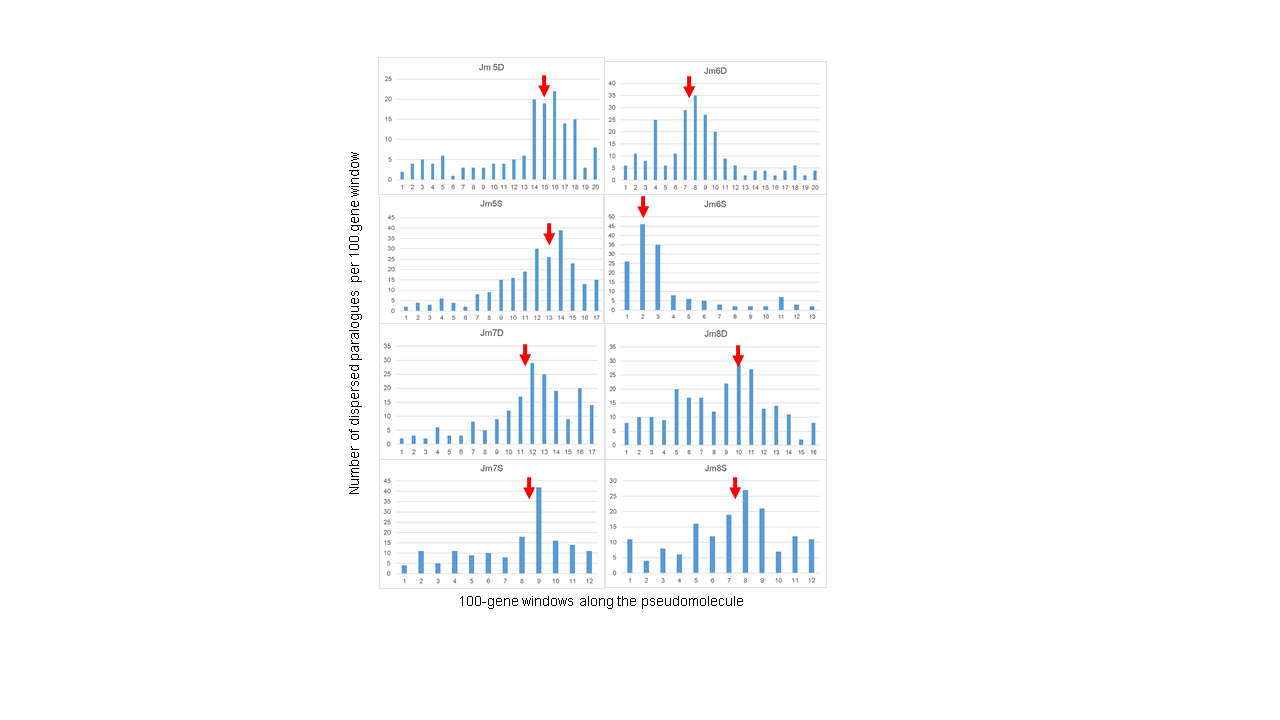 |
| --- |
| **Fig. S17.** **The distribution of dispersed paralogous genes along the *Juglans microcarpa* chromosomes**. The chromosome locations of top hits from BLASTP searches with genes annotated on the JrSerr_v1.0 pseudomolecules (query) against the genes annotated in the Jm31.01_v1.0 pseudomolecules (targets) were recorded and those that were on pseudomolecules other than the corresponding (orthologous) pseudomolecule in Jm31.01_v1.0 were considered as dispersed paralogous genes and counted. Their numbers per non-overlapping windows of 100 query genes were used to construct a histogram. The vertical axis in each histogram is the count of dispersed paralogous genes and the horizontal axis are the 100-gene intervals. The starts of the pseudomolecules are to the left. The red arrows indicate the locations of the centromeric *JCR* arrays. The lengths of the subdominant chromosomes were expanded to equal the length of the dominant chromosome to make the comparison of homoeologues meaningful. |

**Table S1.** Reads produced with the PacBio SMRT cell and 10X Genomics sequencing platforms for hybrid MS1-56

|  | PacBio | | 10X Genomics |
| --- | --- | --- | --- |
|  | **Raw reads** | **Qualified reads** | **Raw reads** |
| Reads (no.) | 4.47 M | 2.05 M | 680.53 M |
| Total length (Gb) | 57.2 | 43.8 |  |
| Mean read length (bp) | 12,791 | 21,322 | 138 |
| Reads N50 (bp) | 19,293 | 22,462 |  |
| Longest read (bp) | 82,702 | 82,702 |  |
| Shortest read (bp) | 35 | 12,000 |  |
| Coverage | 54x | 41x | 89x |

**Table S2.** MS1-56 PacBio sequence assembly and scaffolding

|  | MS1-56_v0 | MS1-56_v1 | MS1-56_scf |
| --- | --- | --- | --- |
| Contig/scaffold (no.) | 460 | 465 | 264 |
| Contig/scaffold N50 (bp) | 7,963,037 | 7,904,778 | 34,776,948 |
| Contig/scaffold max length (bp) | 25,445,311 | 25,445,311 | 52,000,337 |
| Contig/scaffold min length (bp) | 5,379 | 5,379 | 5,379 |
| Assembly total length (bp) | 1,056,053,408 | 1,056,053,408 | 1,066,408,726 |
| N% | 0 | 0 | 0.97 |
| GC% | 36.20 | 36.20 | 35.85 |

**Table S3.** Characteristics of raw molecules and optical maps assemblies of *Juglans regia* Serr, *J. microcarpa* 31.01, and their hybrid MS1-56

|  | MS1-56 | MS1-56 | Serr | 31.01 |
| --- | --- | --- | --- | --- |
| Nickase | Nt.*Bsp*QI | Nb.*Bss*SI | Nt.*Bsp*QI | Nt.*Bsp*QI |
| Molecules (no.) | 529,621 | 537,593 | 474,899 | 550,870 |
| Molecule N50 (Kb) | 297 | 283 | 280 | 321 |
| Molecule min. length (Kb) | 180 | 180 | 180 | 180 |
| Molecule total length (Gb) | 157 | 153 | 134 | 175 |
| Coverage | 135x | 131x | 186x | 295x |
| Contigs (no.) | 624 | 1,276 | 392 | 638 |
| Map total length (Mb) | 1,058 | 1,022 | 642 | 558 |
| Map N50 (Mb) | 2.73 | 1.14 | 2.90 | 1.31 |

**Table S4.** The lengths across the optical maps of *Juglans regia* Serr and *J. microcarpa* 31.01 and the length of the optical map of their hybrid MS1-56

| Optical map | Total length (Mb) | Length of redundant (homologous) contigs (Mb) | Actual length* (Mb) |
| --- | --- | --- | --- |
| Serr | 642 | 108 | 534 |
| 31.01 | 558 | 33 | 525 |
| Serr + 31.01 | 1,200 | 141 | 1,059 |
| MS1-56 | 1,058 | N/A | 1,058 |

*Actual length is the total optical map length (column 2) minus the sum of the redundant (homologous) contigs in regions in which the optical map was diploid (column 3)

**Table S5.** Summary of final assemblies JrSerr_v1.0 and Jm31.01_v1.0

| Chromosome | JrSerr_v1.0 | | | Jm31.01_v1.0 | | |  |
| --- | --- | --- | --- | --- | --- | --- | --- |
|  | **Length (bp)** | **No. of gaps** | **Ns** | **Length (bp)** | **No. of gaps** | **Ns** | |
| chr1 | 43,955,564 | 2 | 0.338% | 43,614,719 | 3 | 0.726% | |
| chr2 | 37,991,177 | 3 | 0.282% | 38,364,941 | 6 | 1.785% | |
| chr3 | 33,932,481 | 2 | 0.113% | 35,798,223 | 3 | 1.495% | |
| chr4 | 32,184,663 | 2 | 0.122% | 32,113,588 | 5 | 2.829% | |
| chr5 | 22,705,590 | 3 | 0.640% | 21,889,424 | 1 | 0.302% | |
| chr6 | 35,197,335 | 3 | 0.208% | 36,225,362 | 2 | 0.374% | |
| chr7 | 51,893,103 | 6 | 0.469% | 49,856,174 | 6 | 0.429% | |
| chr8 | 30,027,985 | 7 | 5.380% | 27,577,207 | 7 | 0.910% | |
| chr9 | 24,894,756 | 3 | 0.738% | 22,373,256 | 1 | 0.140% | |
| chr10 | 37,319,954 | 5 | 0.788% | 34,707,362 | 5 | 0.888% | |
| chr11 | 35,878,018 | 2 | 0.064% | 35,629,462 | 2 | 0.136% | |
| chr12 | 29,818,788 | 1 | 0.226% | 30,169,828 | 4 | 0.453% | |
| chr13 | 38,540,051 | 4 | 0.128% | 39,900,371 | 4 | 0.209% | |
| chr14 | 29,017,359 | 7 | 2.394% | 26,765,518 | 1 | 0.606% | |
| chr15 | 19,617,009 | 3 | 1.684% | 19,962,766 | 2 | 0.116% | |
| chr16 | 28,458,890 | 0 | 0 | 27,832,763 | 2 | 1.457% | |
| Un | 3,239,443 | 1 | 0.02% | 5,115,089 | 0 | 0 | |
| Total | 534,672,166 | 54 | 0.76% | 527,896,053 | 54 | 0.82% | |

**Table S6.** Repeated DNA in the final assemblies JrSerr_v1.0 and Jm31.01_v1.0

|  | JrSerr_v1.0 | | | Jm31.01_v1.0 | |
| --- | --- | --- | --- | --- | --- |
|  | **Length (bp)** | **% of the genome** | **Length (bp)** | | **% of the genome** |
| Class I |  |  |  | |  |
| LTR/Gypsy | 41,269,639 | 7.72 | 46,971,081 | | 8.90 |
| LTR/Copia | 30,025,367 | 5.62 | 31,919,518 | | 6.05 |
| Non-LTR/LINE | 36,776,324 | 6.88 | 30,071,668 | | 5.70 |
| Non-LTR/SINE | 745,329 | 0.14 | 697,079 | | 0.13 |
| Other | 18,493,111 | 3.45 | 20,003,962 | | 3.78 |
| Class II |  |  |  | |  |
| CMC-EnSpm | 9,709,302 | 1.82 | 12,182,151 | | 2.31 |
| hAT-Ac | 7,768,873 | 1.45 | 6,967,092 | | 1.32 |
| MULE-MuDR | 2,879,928 | 0.54 | 1,619,945 | | 0.31 |
| Other | 18,824,118 | 3.52 | 17,485,686 | | 3.31 |
| Unclassified | 52,843,247 | 9.88 | 47,762,358 | | 9.05 |
| Total interspersed repeats | **219,335,238** | **41.02** | **215,680,540** | | **40.86** |
| Satellites | 156,631 | 0.03 | 149,506 | | 0.03 |
| Simple repeats | 15,598,146 | 2.92 | 14,003,109 | | 2.65 |
| Low complexity | 1,810,417 | 0.32 | 1,837,563 | | 0.35 |
| Total | **236,055,467** | **44.15** | **231,618,788** | | **43.88** |

**Table S7.** Simple sequence repeats (SSRs) in the final assemblies JrSerr_v1.0 and Jm3101_v1.0

| SSR motif | Minimum repeats | JrSerr_v1.0 | | Jm3101_v1.0 | |
| --- | --- | --- | --- | --- | --- |
|  |  | **SSR (no.)** | **%** | **SSR (no.)** | **%** |
| Mononucleotide | 10 | 207,790 | 59.14 | 209,883 | 59.98 |
| Dinucleotide | 6 | 118,376 | 33.69 | 115,948 | 33.13 |
| Trinucleotide | 5 | 18,218 | 5.19 | 17,947 | 5.13 |
| Tetranucleotide | 5 | 3,809 | 1.08 | 3,701 | 1.06 |
| Pentanucleotide | 5 | 2,539 | 0.72 | 2,038 | 0.58 |
| Hexanucleotide | 5 | 627 | 0.18 | 434 | 0.12 |
| Total (no.) |  | 351,359 |  | 349,951 |  |
| Total (bp) |  | 661.15 |  | 669.40 |  |

**Table S8**. Characteristics of high-confidence genes annotated in the final JrSerr_v1.0 and Jm3101_v1.0 assemblies

| Category | JrSerr_v1.0 | Jm3101_v1.0 |
| --- | --- | --- |
| Genes (no.) | 31,425 | 29,496 |
| Exons (no.) | 170,804 | 165,316 |
| CDS (no.) | 164,444 | 161,065 |
| Five prime UTRs (no.) | 15,290 | 9,827 |
| Three prime UTRs (no.) | 15,920 | 11,379 |
| Average gene length(bp) | 4,226 | 4,391 |
| Exons per gene (no.) | 5.44 | 5.60 |
| CDSs per gene (no.) | 5.23 | 5.46 |
| CDS length per gene (bp) | 1,173.00 | 1,240.00 |
| CDS length (bp) | 224.14 | 227.05 |
| Exon length (bp) | 258.64 | 249.38 |

**Table S9.** High-confidence genes in the pseudomolecules and unassigned (Un) scaffolds in the Serr assembly JrSerr_v1.0 and 31.01 assembly Jm31.01_v1.0

| JrSerr_v1.0 | | | | Jm31.01_v1.0 | | | |
| --- | --- | --- | --- | --- | --- | --- | --- |
| Pseudomol. | **Genes (no.)** | **Pseudomol. length (bp)** | **Gene density (genes/Mb)** | **Pseudomol.** | **Genes (no.)** | **Pseudomol. length (bp)** | **Gene density (genes/Mb)** |
| Jr1 | 2,662 | 43,955,564 | 60.56 | **Jm1** | 2,581 | 43,614,719 | 59.18 |
| Jr2 | 2,088 | 37,991,167 | 54.96 | **Jm2** | 1,922 | 38,364,907 | 50.10 |
| Jr3 | 2,120 | 33,932,481 | 62.48 | **Jm3** | 2,069 | 35,798,223 | 57.80 |
| Jr4 | 1,792 | 32,184,663 | 55.68 | **Jm4** | 1,654 | 32,113,326 | 51.51 |
| Jr5 | 1,401 | 22,705,590 | 61.70 | **Jm5** | 1,267 | 21,889,424 | 57.88 |
| Jr6 | 1,678 | 35,197,335 | 47.67 | **Jm6** | 1,543 | 36,225,362 | 42.59 |
| Jr7 | 3,076 | 51,893,103 | 59.28 | **Jm7** | 2,788 | 49,856,174 | 55.92 |
| Jr8 | 1,722 | 30,027,985 | 57.35 | **Jm8** | 1,617 | 27,577,207 | 58.64 |
| Jr9 | 1,420 | 24,894,769 | 57.04 | **Jm9** | 1,293 | 22,373,256 | 57.79 |
| Jr10 | 1,995 | 37,319,718 | 53.46 | **Jm10** | 1,913 | 34,707,362 | 55.12 |
| Jr11 | 2,294 | 35,878,018 | 63.94 | **Jm11** | 2,187 | 35,629,462 | 61.38 |
| Jr12 | 1,885 | 29,818,788 | 63.22 | **Jm12** | 1,839 | 30,169,828 | 60.95 |
| Jr13 | 2,437 | 38,540,051 | 63.23 | **Jm13** | 2,360 | 39,900,371 | 59.15 |
| Jr14 | 1,824 | 29,017,359 | 62.86 | **Jm14** | 1,643 | 26,765,518 | 61.38 |
| Jr15 | 1,135 | 19,617,005 | 57.86 | **Jm15** | 1,073 | 19,962,766 | 53.75 |
| Jr16 | 1,757 | 28,458,890 | 61.74 | **Jm16** | 1,648 | 27,832,763 | 59.21 |
| JrUn | 139 |  | NA | **JmUn** | 99 |  | NA |
| Total | 31,425 |  |  | **Total** | 29,496 |  |  |

**Table S10.** Assessment of completeness of gene annotation in our *Juglans* genome assemblies and a published assembly (Chandler) with the BUSCO v3.0 gene set

| BUSCO gene status | JrSerr_v1.0 | Jm31.01_v1.0 | *J. regia* Chandler* |
| --- | --- | --- | --- |
| Complete and single-copy (S) | 1,201 (83.4%) | 1,201 (83.4%) | 1,167 (81.0%) |
| Complete and duplicated (D) | 160 (11.1%) | 148 (10.3%) | 199 (13.8%) |
| Fragmented (F) | 22 (1.5%) | 22 (1.5%) | 17 (1.2%) |
| Missing (M) | 57 (4.0%) | 69 (4.8%) | 57 (4.0%) |
| Total | 1,440 | 1,440 | 1,440 |

*From ^2^.

**Table S11.** Transcription factors (TFs) annotated in the final JrSerr_v1.0 and Jm31.01_v1.0 assemblies

| Category of TF | JrSerr_v1.0 | Jm31.01_v1.0 |
| --- | --- | --- |
| MYB | 181 | 154 |
| bHLH | 154 | 160 |
| AP2/ERF-ERF | 147 | 140 |
| C2H2 | 143 | 147 |
| NAC | 120 | 134 |
| MYB-related | 106 | 111 |
| WRKY | 83 | 81 |
| bZIP | 74 | 77 |
| GRAS | 74 | 74 |
| C3H | 73 | 63 |
| FAR1 | 56 | 48 |
| GARP-G2-like | 52 | 54 |
| HB-HD-ZIP | 48 | 50 |
| LOB | 46 | 48 |
| Trihelix | 44 | 44 |
| C2C2-Dof | 43 | 47 |
| B3 | 39 | 45 |
| C2C2-GATA | 31 | 30 |
| SBP | 31 | 32 |
| TCP | 29 | 29 |
| B3-ARF | 26 | 26 |
| MADS-MIKC | 26 | 23 |
| OFP | 25 | 23 |
| HSF | 23 | 14 |
| MADS-M-type | 23 | 27 |
| Tify | 19 | 19 |
| NF-YB | 18 | 21 |
| PLATZ | 18 | 20 |
| HB-WOX | 17 | 17 |
| AP2/ERF-AP2 | 16 | 17 |
| HB-BELL | 16 | 19 |
| HB-other | 14 | 15 |
| NF-YC | 13 | 11 |
| C2C2-CO-like | 12 | 11 |
| NF-YA | 12 | 12 |
| TUB | 12 | 12 |
| zf-HD | 12 | 13 |
| RWP-RK | 11 | 11 |
| SRS | 11 | 13 |
| BBR-BPC | 10 | 9 |
| C2C2-YABBY | 10 | 10 |
| GARP-ARR-B | 10 | 10 |
| BES1 | 9 | 10 |
| HB-KNOX | 9 | 5 |
| E2F-DP | 8 | 7 |
| EIL | 8 | 7 |
| LIM | 8 | 6 |
| CAMTA | 7 | 5 |
| CPP | 7 | 7 |
| GRF | 7 | 8 |
| Alfin-like | 6 | 8 |
| GeBP | 6 | 6 |
| DBB | 5 | 7 |
| DBP | 5 | 5 |
| AP2/ERF-RAV | 4 | 6 |
| C2C2-LSD | 4 | 4 |
| ULT | 4 | 2 |
| HB-PHD | 3 | 2 |
| VOZ | 3 | 3 |
| Whirly | 3 | 2 |
| CSD | 2 | 1 |
| NF-X1 | 2 | 2 |
| S1Fa-like | 2 | 2 |
| BSD | 1 | 1 |
| HRT | 1 | 1 |
| LFY | 1 | 1 |
| NOZZLE | 1 | 1 |
| SAP | 1 | 1 |
| STAT | 1 | 1 |

**Table S12.** *K*a and *K*s values between paralogous genes identified by bidirectional BLASTP on the homoeologous chromosomes within the *Juglans regia* and *J. microcarpa* genomes

| Homoeologous pair* | *Jr* genes | *Jm* genes | *Jr K*a | *Jm K*a | *Jr K*s | *Jm K*s |
| --- | --- | --- | --- | --- | --- | --- |
| Jr1- Jr10 | 613 | 634 | 0.0735 | 0.0781 | 0.3218 | 0.3226 |
| Jr2-Jr9 | 442 | 430 | 0.0629 | 0.0722 | 0.3245 | 0.3301 |
| Jr3-Jr4 | 523 | 482 | 0.0702 | 0.0696 | 0.3342 | 0.3257 |
| Jr14-Jr5 | 372 | 371 | 0.0774 | 0.0776 | 0.3249 | 0.3297 |
| Jr6-Jr15 | 261 | 255 | 0.0817 | 0.0724 | 0.3403 | 0.3319 |
| Jr7-Jr12 | 697 | 667 | 0.0639 | 0.0635 | 0.3227 | 0.3296 |
| Jr11-Jr8 | 491 | 487 | 0.0884 | 0.0839 | 0.3346 | 0.3335 |
| Jr13-Jr16 | 571 | 550 | 0.0697 | 0.0687 | 0.3298 | 0.3208 |
| Mean (total) | (3,970) | (3,876) | 0.0735 | 0.0733 | 0. 3291 | 0.3280 |
| Standard error |  |  | 0.0169 | 0.0124 | 0.0130 | 0.0871 |

*The first chromosome in a pair is the dominant chromosome.

**Table S13** *K*s and *Ka* values between orthologous *Juglans regia,* *J. microcarpa*, and grape (*Vitis vinifera*) genes and paralogous genes within the *J. regia* and *V. vinifera* genomes that evolved by the Juglandoid WGD and the γWGT, respectively, and the estimation of divergence times

| Compared genomes | Genes | *K*a | Divergence (MY)* | *K*s | Divergence (MY)* |
| --- | --- | --- | --- | --- | --- |
| *J. regia - J. microcarpa** | Orthologs | 0.0089 | 8.0 (6.9-9.7) | 0.0299 | 6.0 (5.8-6.1) |
| *J. regia* - *J. microcarpa*** | Orthologs | 0.0076 | 6.8 (5.7-8.4) | 0.0304 | 6.1 (5.9-6.3) |
| *J. regia* - *J. regia** | Paralogs | 0.0735 | 66 (55.3-81.7) | 0.3291 | 66 (63.9-68.3) |
| *J. microcarpa - J. microcarpa** | Paralogs | 0.0733 | 66.0 (57.8-76.9.2) | 0.3280 | 66.0 (64.6-67.5) |
| *J. regia* - *V. vinifera* | Orthologs | 0.1529 | 137.3 (115.0-169.9) | 0.7039 | 141.2 (136.7-146.1) |
| *V. vinifera - V. vinifera* | Paralogs | 0.1931 | 173.4 (145.3-214.6) | 0.89 | 179.0 (175.1-183.1) |
| *J. regia – A. trichopoda* | Orthologs | 0.2536 | 227.7 (191.0-282.0) | 1.3767 | 276.1 (267.2-285.5) |

*These values are from Table S12.

**Based on analyses of the sequences of 1077 *J. regia* Chandler and *J. microcarpa* acc. DJUG 29.11 orthologues genes ^3^.

**Table S14**. Numbers of major chromosome rearrangements and the rates of their accumulation per MY in the phylogenetic branches of since the Juglandoid WGD to the present

| Branch | Abbrev. | Time (MY) | Major rear. (no) | Rate (rear./MY) |
| --- | --- | --- | --- | --- |
| Prior to Jr-Jm divergence | RM | 57 | 25 | 0.4 |
| *J. regia* (Jr) | R | 9 | 5 | 0.6 |
| *J. microcarpa* (Jm) | M | 9 | 13 | 1.4 |

**Table S15.** Correspondence of the *Juglans regia* chromosomes to grape (Vv) synteny blocks

| Chromosome | Synteny blocks in the grape genome |
| --- | --- |
| 1D | Vv7, Vv4, (Vv13)*, Vv4, Vv15, (Vv9), Vv15, Vv5(cen)**, Vv9, Vv15, Vv13 |
| 1S | Vv7, Vv4, Vv15, cen***, Vv5, Vv9, Vv15, Vv13 |
| 2D | Vv14, Vv16, Vv14, Vv12(cen), Vv11, Vv7, Vv12 |
| 2S | Vv14, Vv16, Vv3, Vv7, (Vv4), Vv7, Vv12, cen, Vv11, Vv7 |
| 3D# | Vv12, Vv3, Vv12, Vv3, Vv1(cen), Vv16, (Vv18), Vv16, (Vv1), Vv16 |
| 3S# | Vv16, (Vv1), Vv16, (Vv18), Vv16, Vv1(cen), Vv3, (Vv12), Vv3, Vv12 |
| 4D# | Vv7, Vv4, Vv14, (Vv4), Vv14, Vv1, Vv4(cen), Vv7, Vv18, Vv11, (Vv4), Vv11, Vv4 |
| 4S# | Vv4, Vv11~~,~~ Vv18, Vv7, cen, Vv4, (Vv14), Vv4, (Vv7), Vv4 |
| 5D | Vv8, Vv10, (Vv1), Vv10, (Vv1), Vv10, Vv8(cen) |
| 5S | Vv8, Vv10, (Vv1), Vv10, Vv1, cen, Vv8, (Vv12), Vv8, Vv12 |
| 6D | Vv17, Vv19, cen, Vv2, Vv19 |
| 6S | Vv17(cen), Vv2, Vv19 |
| 7D | Vv13, (Vv15), Vv13, Vv6(cen) |
| 7S | Vv13, (Vv15), Vv13, Vv3, Vv6(cen) |
| 8D | Vv10, Vv1, Vv18, (Vv4), Vv18, (Vv1), Vv18, Vv4, cen Vv16, Vv19, Vv17, Vv1, Vv18, Vv11, Vv7 |
| 8S | Vv10, Vv1, Vv18, (Vv4), Vv18, (Vv1), Vv18, Vv4(cen), Vv16, Vv19, Vv17, (Vv1), Vv17, Vv1, Vv18, Vv11, Vv7. Vv11, Vv7 |

*Synteny blocks in parentheses are interstitial translocations.

**Indicates centromeres located within a synteny block.

***Indicates centromere located between synteny blocks.

^#^Homoeologous pseudomolecules are inverted relative to each other.

**Table S16**. Percentages of collinear genes in comparisons of genomes in woody perennials and grasses

| Divergence | Time (MY) | Collinear genes (%) |
| --- | --- | --- |
| *Juglans regia* vs *Juglans microcarpa* | 8 | 88.4 |
| *J. regia* vs *Vitis vinifera* | 137 | 43.7 |
| *Aegilops tauschii* vs wheat A genome | 3 | 50.0 |
| *Ae. tauschii* vs *Oryza sativa* | 47 | 34.6 |
| *O. sativa* vs *Ae. tauschii* | 47 | 32.2 |
| *O. sativa* vs *Brachypodium distachyon* | 47 | 40.1 |
| *O. sativa* vs *Sorghum bicolor* | 55 | 39.3 |

**Table S17.** Renaming of *Juglans regia* and *J. microcarpa* dominant and subdominant chromosomes based on homoeologous relationships and the number of genes annotated in the dominant chromosome

| Dominant chromosome* | *J. regia* | *J. microcarpa* | Subdominant homoeologue | *J. regia* | *J. microcarpa* |
| --- | --- | --- | --- | --- | --- |
| 7 | Jr1D | Jm1D | **12** | Jr1S | Jm1S |
| 1 | Jr2D | Jm2D | **10** | Jr2S | Jm2S |
| 13 | Jr3D | Jm3D | **16 (inverted)*** | Jr3S | Jm3S |
| 11 | Jr4D | Jm4D | **8 (inverted)*** | Jr4S | Jm4S |
| 3 | Jr5D | Jm5D | **4** | Jr5S | Jm5S |
| 2 | Jr6D | Jm6D | **9** | Jr6S | Jm6S |
| 14 | Jr7D | Jm7D | **5** | Jr7S | Jm7S |
| 6 | Jr8D | Jm8D | **15** | Jr8S | Jm8S |

*****The homoeologous pseudomolecules are inverted relative to each other.

**Table S18.** Numbers of genes on the JrSerr_v1.0 and Jm31.01_v1.0 dominant and subdominant pseudomolecules

| Dominant | Serr | 31.01 | Subdominant | Serr | 31.01 |
| --- | --- | --- | --- | --- | --- |
| Jr1 | 2,662 | 2,581 | **Jr10** | 1,995 | 1,913 |
| Jr2 | 2,088 | 1,922 | **Jr9** | 1,420 | 1,293 |
| Jr3 | 2,120 | 2,069 | **Jr4** | 1,792 | 1,654 |
| Jr14 | 1,824 | 1,643 | **Jr5** | 1,401 | 1,267 |
| Jr6 | 1,678 | 1,543 | **Jr15** | 1,135 | 1,073 |
| Jr7 | 3,076 | 2,788 | **Jr12** | 1,885 | 1,839 |
| Jr11 | 2,294 | 2,187 | **Jr8** | 1,722 | 1,617 |
| Jr13 | 2,437 | 2,360 | **Jr16** | 1,757 | 1,648 |
| Total | 18,179 | 17,093 |  | 13,107 | 12,304 |
| Mean* | 2,272 | 2,137 |  | 1,638 | 1,538 |

**P*=0.0002 and 3.9E-05 for the mean number of genes in the dominant and subdominant chromosomes in Serr and 31.01, respectively.

**Table S19.** Numbers of singleton genes in the intervals between successive collinear gene pairs in dominant and subdominant homoeologous pseudomolecules in the JrSerr_v1.0 genome sequence

| Homoeologous pair* | Collinear gene pairs | Singletons in dominant pseud. | Singletons in subdominant pseud. | *P* |
| --- | --- | --- | --- | --- |
| Jr1D-Jr1S | 752 | 2,320 | 1,090 | 4.2E-16 |
| Jr2D- Jr2S | 640 | 2,008 | 1,283 | 8.8E-10 |
| Jr3D-Jr3S | 596 | 1,839 | 1,056 | 2.9E-08 |
| Jr4D-Jr4S | 562 | 1,705 | 1,089 | 4.7E-04 |
| Jr5D-Jr5S | 569 | 1,539 | 1,128 | 6.8E-04 |
| Jr6D-Jr6S | 472 | 1,609 | 880 | 2.4E-06 |
| Jr7D-Jr7S | 409 | 1,393 | 943 | 5.7E-05 |
| Jr8D-Jr8S | 293 | 1,378 | 792 | 1.9E-06 |
| Total | 4,293 | 13,791 | 8,261 | 1.2E-04 |

*The first chromosome in the pair is the dominant chromosome.

**Table S20.** Tissues, developmental stages, and sources of cDNA libraries used for RNAseq analyses of gene expression on dominant and subdominant homoeologues in the walnut genome

| cDNA library serial number | Tissue | Developmental stage/Source | Abbreviation |
| --- | --- | --- | --- |
| 1 | Pellicle | Early stage/Chandler | CHE |
| 2 | Pellicle | Middle stage/Chandler | CHM |
| 3 | Callus exterior | Vegetative/Chandler | CE |
| 4 | Callus interior | Vegetative/Chandler | CI |
| 5 | Catkins | Immature/Chandler | CK |
| 6 | Embryo | Mature/Mixed | EM |
| 7 | Pistillate flower | Vegetative/Chandler | FL |
| 8 | Hull cortex | Mature/Chandler | HC |
| 9 | Hull | Immature/Chandler | HI |
| 10 | Hull peel | Mature/Chandler | HP |
| 11 | Hull – dehiscing | Senescent/Chandler | HU |
| 12 | Fruit | Immature/Mixed | IF |
| 13 | Leaves | Vegetative/Chandler | LE |
| 14 | Leaf – mature | Vegetative/Chandler | LM |
| 15 | Leaf – early | Vegetative/Chandler | LY |
| 16 | Packing tissue | Mature/Chandler | PK |
| 17 | Pellicle | Mature/Chandler | PL |
| 18 | Packing tissue | Immature/Chandler | PT |
| 19 | Root | Vegetative/Chandler | RT |
| 20 | Somatic embryo | Immature/Chandler | SE |
| 21 | Transition wood | Transition zone/*J. nigra* | TZ |
| 22 | Vegetative bud | Vegetative/Chandler | VB |

**Table S21.** Numbers of telomeric repeats in the most distal 10 Kb of a chromosome arm and the length of the missing telomeric region inferred from the optical maps of Serr and 31.01

| Serr chrom. arm | Telom. repeats (present?) | Repeats (no.) | Missing (bp) | 31.01 chrom.arm | Telom. repeats (present?) | Repeats (no.) | Missing (bp) |
| --- | --- | --- | --- | --- | --- | --- | --- |
| Jr1Dp | Y | 739 |  | Jm1Dp | N | - | 6,590 |
| Jr1Dq | Y | 487 |  | Jm1Dq | N | - | - |
| Jr2Dp | N | - | 12,709 | Jm2Dp | Y | 898 |  |
| Jr2Dq | N | - | 17,930 | Jm2Dq | N | - | - |
| Jr3Dp | N | - | 23,936 | Jm3Dp | Y | 1,211 |  |
| Jr3Dq | N | - | 3,183 | Jm3Dq | Y | 1,051 |  |
| Jr4Dp | N | - |  | Jm4Dp | Y | 1,028 |  |
| Jr4Dq | N | - | 10,028 | Jm4Dq | N | - | 2,943 |
| Jr5Dp | Y | 770 |  | Jm5Dp | Y | 649 |  |
| Jr5Dq | N | - | 14,612 | Jm5Dq | N | - | 2,457 |
| Jr6Dp | Y | 520 |  | Jm6Dp | N | - | 862 |
| Jr6Dq | Y | 496 |  | Jm6Dq | N | - | - |
| Jr7Dp | Y | 618 |  | Jm7Dp | N | - | 12,859 |
| Jr7Dq | Y | 784 |  | Jm7Dq | N | - | - |
| Jr8Dp | N | - | - | Jm8Dp | Y | 926 |  |
| Jr8Dq | Y | 594 |  | Jm8Dq | N | - | 12,771 |
| Jr1Sp | N | - | 12,292 | Jm1Sp | N | - | 8,851 |
| Jr1Sq | Y | 1,050 |  | Jm1Sq | Y | 914 |  |
| Jr2Sp | N | - | 8,466 | Jm2Sp | N | - | 12,404 |
| Jr2Sq | Y | 810 |  | Jm2Sq | N | - |  |
| Jr3Sp | N | - | 3,555 | Jm3Sp | N | - | 50 |
| Jr3Sq | N | - | 4,785 | Jm3Sq | Y | 1,224 |  |
| Jr4Sp | Y | 883 |  | Jm4Sp | N | - | - |
| Jr4Sq | N | - | 8,839 | Jm4Sq | N | - | 5,701 |
| Jr5Sp | Y | 1,012 |  | Jm5Sp | N | - | 3,904 |
| Jr5Sq | Y | 895 |  | Jm5Sq | Y | 787 |  |
| Jr6SS | N | - | - | Jm6SS | N | - | 19,664 |
| Jr6Sq | Y | 2,051 |  | Jm6Sq | Y | 929 |  |
| Jr7Sp | N | - | 33,900 | Jm7Sp | Y | 1,186 |  |
| Jr7Sq | Y | 793 |  | Jm7Sq | N | - | 53,154 |
| Jr8Sp | N | - | 950 | Jm8Sp | N | - | 5,123 |
| Jr8Sq | Y | 862 |  | Jm8Sq | N | **-** | **-** |

**Table S22. Summary of RGA identification results for *J. regia* and *J. microcarpa***

| R category | | JrSerr_v1.0 | Jm31.01_v1.0 |
| --- | --- | --- | --- |
| NBS encoding | NBS | 10 | 8 |
|  | CNL | 32 | 27 |
|  | TNL | 3 | 16 |
|  | CN | 8 | 9 |
|  | TN | 1 | 3 |
|  | NL | 22 | 18 |
|  | TX | 13 | 17 |
|  | Others | 3 | 5 |
| RLP | | 78 | 68 |
| RLK | | 585 | 528 |
| TM-CC | | 187 | 204 |
| Total | | 942 | 903 |

CC: Coiled-coil; CN: CC-NBS; LRR: Leucine rich repeat; NBS: Nucleotide-binding site; RLK: Receptor like kinase; RLP: Receptor like protein; STK: Serine-threonine kinase; TIR: Toll/interleukin-1 receptor; TM: Transmembrane; CNL: CC-NBS-LRR; NL: NBS-LRR; TN: TIR-NBS; TNL: TIR-NBS-LRR; TX: TIR-unknown domain.

**SI References**

1. Kumar, S., Stecher, G., Li, M., Knyaz, C. & Tamura, K. MEGA X: Molecular Evolutionary Genetics Analysis across computing platforms. *Mol. Biol. Evol.* **35**, 1547-1549 (2018).

2. Martinez-Garcia, P.J. et al. The walnut (*Juglans regia*) genome sequence reveals diversity in genes coding for the biosynthesis of non-structural polyphenols. *Plant J.* **87**, 507-532 (2016).

3. Stevens, K.A. et al. Genomic variation among and within six *Juglans* species. *G3: Genes|Genomes|Genetics* **8**, doi:10.1534/g3.118.200030 (2018).
